# Fossilized Melts in Mantle Wedge Peridotites

**DOI:** 10.1038/s41598-018-28264-6

**Published:** 2018-07-04

**Authors:** Kosuke Naemura, Takao Hirajima, Martin Svojtka, Ichiko Shimizu, Tsuyosi Iizuka

**Affiliations:** 1 Nagoya University Museum, Furo-cho, Chikusa-ku, Nagoya 464-8601 Japan; 2 Department of Earth and Planetary Science, Graduate School of Science, The University of Tokyo, 7-3-1 Hongo, Bunkyo-ku, Tokyo 113-0033 Japan; 3 Division of Earth and Planetary Sciences, Graduate School of Science, Kyoto University, Kitashirakawa Oiwake-cho, Sakyo-ku, Kyoto 606-8502 Japan; 4 Institute of Geology of the Czech Academy of Sciences, Rozvojová 269, 165 00, Praha - Lysolaje, Czech Republic

**Keywords:** Geochemistry, Petrology

## Abstract

The shallow oxidized asthenosphere may contain a small fraction of potassic silicate melts that are enriched in incompatible trace elements and volatiles. Here, to determine the chemical composition of such melt, we analysed fossilized melt inclusions, preserved as multiphase solid inclusions, from an orogenic garnet peridotite in the Bohemian Massif. Garnet-poor (2 vol.%) peridotite preserves inclusions of carbonated potassic silicate melt within Zn-poor chromite (<400 ppm) in the clinopyroxene-free harzburgite assemblage that equilibrated within the hot mantle wedge (Stage 1, > 1180 °C at 3 GPa). The carbonated potassic silicate melt, which has a major element oxide chemical composition of K_2_O = 5.2 wt.%, CaO = 17 wt.%, MgO = 18 wt.%, CO_2_ = 22 wt.%, and SiO_2_ = 20 wt.%, contains extremely high concentrations of large ion lithophile elements, similar to kimberlite melts. Peridotites cooled down to ≅800 °C during Stage 2, resulted in the growth of garnet relatively poor in pyrope content, molar Mg/(Mg + Fe + Ca + Mn), (ca. 67 mol.%). This garnet displays a sinusoidal REE pattern that formed in equilibrium with carbonatitic fluid. Subsequently, subduction of the peridotite resulted in the formation of garnet with a slightly higher pyrope content (70 mol.%) during the Variscan subduction Stage 3 (950 °C, 2.9 GPa). These data suggest the following scenario for the generation of melt in the mantle wedge. Primarily, infiltration of sediment-derived potassic carbonatite melt into the deep mantle wedge resulted in the growth of phlogopite and carbonate/diamond. Formation of volatile-bearing minerals lowered the density and strength of the peridotite. Finally, phlogopite-bearing carbonated peridotite rose as diapirs in the mantle wedge to form carbonated potassic silicate melts at the base of the overriding lithosphere.

## Introduction

Subduction of carbonates stored within the oceanic and continental crust at convergent plate boundaries has played an important role in mantle refertilization and the global carbon cycle^1^. The total carbon input to subduction zone systems has been estimated to be as high as 24–48 megatons per year, whereby 17 megatons of crustal carbon are returned to the surface through volcanism^1^. Consequently, a significant quantity of carbon could be stored within the downgoing plate and returned to the deep mantle in the form of high-pressure carbonate minerals^2^. In warm subduction zones and collision zones, partial melting of subducting plate could remove carbon more efficiently from the downgoing crust^3^. Due to their low viscosity and high mobility, carbonate-rich melts (i.e. carbonatites) are expected to percolate into the overlying mantle wedge^4^. Carbonatite not only refertilizes peridotite in the mantle wedge, but also lowers the density of the mantle wedge by precipitating volatile-rich minerals. A reduction in density can generate Rayleigh–Taylor instabilities and initiate diapiric flow^5^. The presence of carbonates, graphite, diamond and carbonate-rich multiphase solid inclusions (MSIs) has been reported in mantle wedge peridotites from ultrahigh-pressure (UHP) metamorphic terranes^6–9^. Such C-bearing phases may provide direct geological evidence for the presence of mantle wedge melts.

Mantle wedge peridotites record polyphase metasomatism during transfer from the asthenosphere into the subducting continental crust^6,7,10^. The initial stages of metasomatism are induced by the infiltration of melts at temperatures (*T*) in excess of 1200 °C, whereas secondary metasomatism results from the addition of crustal fluids at 500–850 °C^6,10^. This study aims to constrain melt compositions in mantle wedge peridotites. Previous studies have characterised melt compositions using trace elements in nominally anhydrous minerals^6,10^ or whole-rock samples^10^. Both methods indicate the presence of high-temperature silicate melts that are enriched in incompatible trace elements such as light-REE (LREE), Pb, U, and Li. Here, we examine fossilized melt inclusions^11^ to constrain the major and trace element compositions of potassium-rich carbonatitic melts in the mantle wedge.

## Mantle wedge peridotites in the Bohemiam Massif

To study melts within the mantle wedge, we investigated an orogenic garnet peridotite from the Moldanubian Zone of the Bohemian Massif (BM) in the Czech Republic, which experienced corner flow in the mantle wedge and contains MSIs (i.e. fossilized melt)^7,12,13^. Orogenic garnet peridotite bodies within the Moldanubian Zone occur as lenticular bodies ranging in size from several centimetres to kilometres across, and are surrounded by felsic granulites and gneisses. A previous study has shown that Mg–Cr-rich garnet peridotites within granulite equilibrated in a low P/T regime (2.5–3.0 GPa, 1015–1335 °C) and were possibly derived from the asthenosphere, whereas those occurring within migmatitic orthogneiss equilibrated in a medium P/T regime (3.3–6.0 GPa, 875–1180 °C) within the lithospheric mantle wedge^14^. The Blanský les granulite^15^ in the BM has a cylindrical dome structure and contains numerous serpentinized garnet peridotite bodies that occur mainly in a wide NW–SE striking serpentinite belt in the central part of the granulite massif. At the SE margin of the serpentinite belt, fresh spinel–garnet peridotite crops out within the Plešovice quarry.

**Figure 1:**
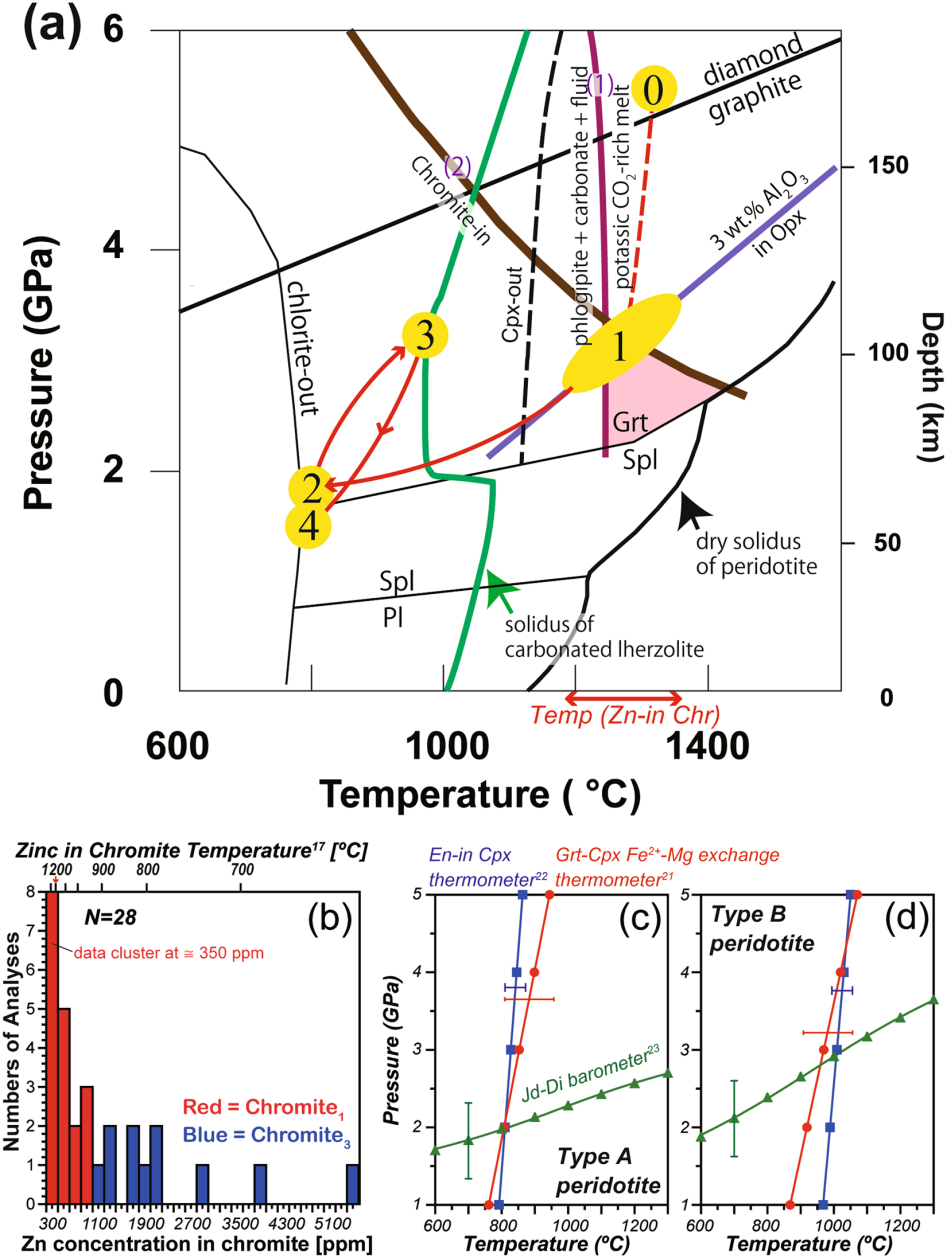
Panel (a) shows a pressure–temperature path of the Plešovice peridotite, which consists of four stages represented by yellow circles. The decarbonation reaction, recording the breakdown of phlogopite + magnesite (1) is from ref.^32^, and the chromite (Chr)-in reaction (2) for depleted lherzolite from ref.^31^. The graphite–diamond transition is from ref.^65^. The solidus for metasomatized peridotite is from ref.^30^ clinopyroxene (Cpx)-out curve is from ref.^20^ other phase boundaries are after ref.^7^ and references therein. Pl = Plagioclase^16^. In panel (b) Zn concentrations of Chr are summarized. Zn concentration of Chr_1_ grains included in garnet (Grt) are 300–950 ppm, and those of matrix Chr_3_ are 1200–5600 ppm. Zinc-in-chromite temperatures^17^ are shown on the upper axis. Panels (c) and (d) are results of thermobarometric calculations for Type A and B peridotites, respectively. The Grt–Cpx thermobarometers of refs^21–23^ have been used.

Figure 1 summarizes the thermal history of the Plešovice peridotite^7,12^, which may have experienced *P–T* conditions within the diamond stability field at the earliest stage (Stage 0, > 4 GPa). The peridotite was then uplifted to shallow depths (Stage 1)^7^ and subsequently cooled into the chlorite stability field (Stage 2, < 750 °C). It was then subducted into the high *P–T* garnet lherzolite field (Stage 3, 950 °C at 2.9 GPa). At the final stage, the peridotite was partially recrystallized to spinel lherzolite assemblage (Stage 4, 750 °C at 1.5 GPa). Previous Sm–Nd geochronological studies have yielded ages of 375 to 329 Ma for the garnet peridotites in the Moldanubian Zone of the BM^14^.

**Figure 2:**
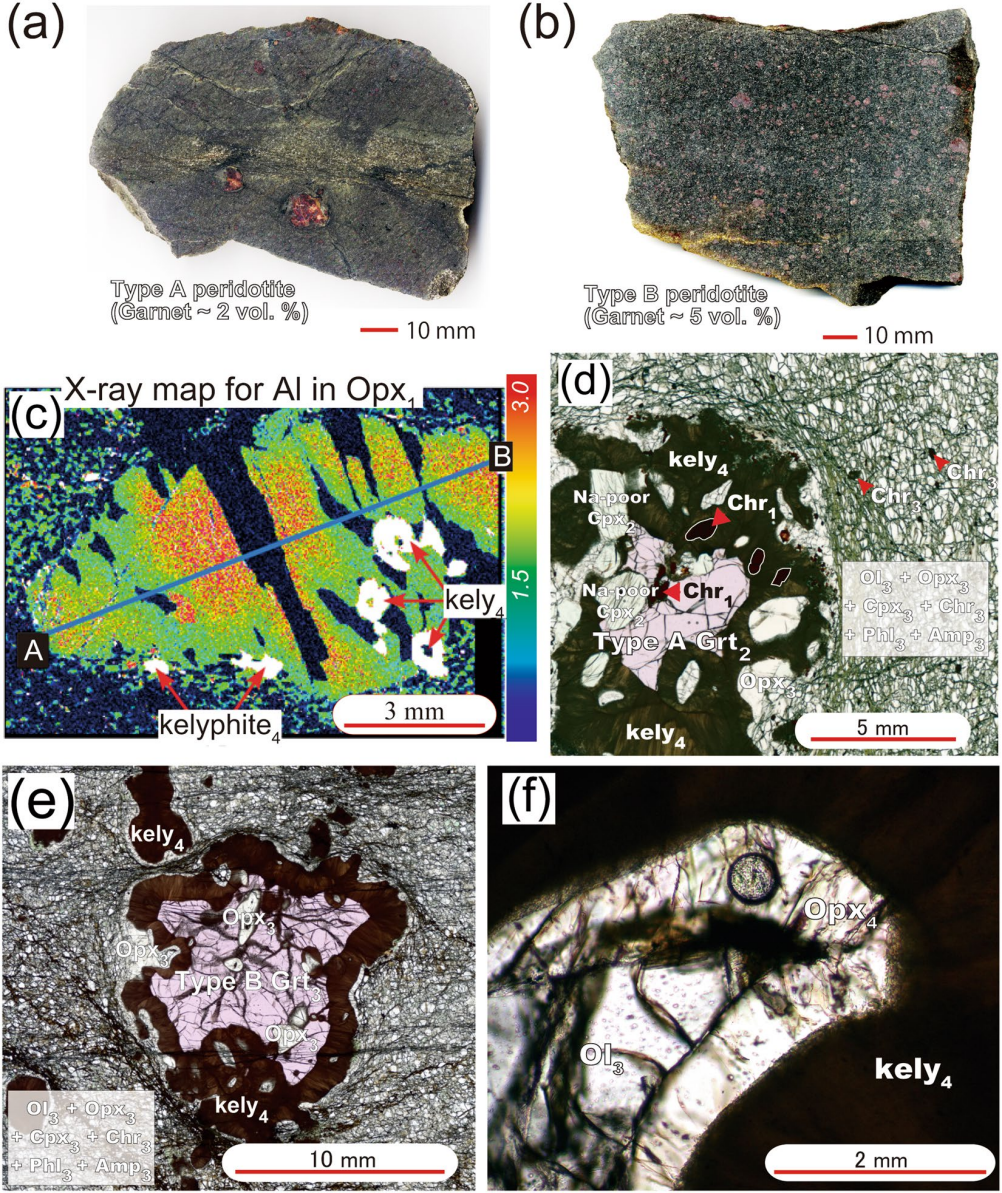
Panels (a) and (b) show typical Type A and B peridotites from the Plešovice quarry in the Bohemian Massif. Panel (c) shows Al_2_O_3_ weight % concentration map of an orthopyroxene porphyroclast (Opx_1_) stable at Stage 1 in a Type A peridotite. Crystallization stages are denoted by subscript numbers in figures. Chemical analyses along the traverse from A to B are shown in ref.^7^. Kelyphite (kely_4_) formed after garnet (Grt). Matrix consists of olivine (Ol_3_), Al-poor orthopyroxene (Opx_3_), Na_2_O-rich clinopyroxene (Cpx_3_), chromite (Chr_3_), phlogopite (Phl_3_), and calcic amphibole (Amp_3_) that formed during Stage 3^7^. Panel (d) displays a centimetre-sized garnet (Grt_2_) with Na_2_O-poor (~0.35 wt.%) Cpx_2_ within embayments in a Type A peridotite. Plane polarized light. Panel (e) shows a Grt_3_ with large Opx_3_ inclusions within a Type B peridotite. Plane polarized light. Panel (f) shows a micrograph of kelyphite, which are separated from matrix Ol_3_ by an orthopyroxene corona (Opx_4_). Plane polarized light.

The spinel–garnet peridotite at Plešovice is a depleted lherzolite consisting mainly of olivine (Ol) and orthopyroxene (Opx) (14–16 vol.%) with minor clinopyroxene (Cpx) (1 vol.%) and calcic amphibole (Amp) (1–2 vol.%) and accessory chromian spinel (Chr), phlogopite (Phl) and apatite (Ap)^7^. Mineral abbreviations follows ref.^16^. Two lithological types of the spinel–garnet peridotite, Type A and Type B, have been distinguished by variations in the abundance of garnet (Grt) (~2 and ~5 vol.%, respectively). Garnet occurs as sporadic large grains (>5 mm) in Type A peridotites, whereas they are ubiquitous and show a range of grain sizes (0.1–30 mm) in Type B peridotites (Fig. 2a,b). We document the trace element composition of the minerals in order to correlate metasomatic events within the existing framework of *P–T* constraints^7,12^.

**Figure 3:**
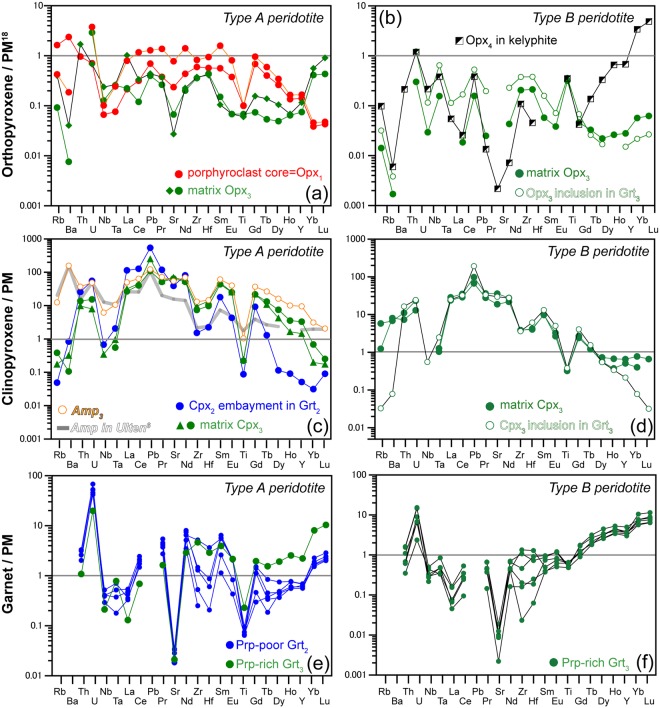
Panels (a,b) show primitive-mantle (PM) normalized^18^ trace element patterns of orthopyroxene (Opx) in Type A and Type B peridotites, with stages are denoted by subscript numbers in the figure. Opx_1_ displays a negative Ti anomaly and is rich in large ion lithophile element (LILE) and light REE (LREE). Opx_3_ in Type A peridotites shows a convex REE pattern with an increase in heavy REE (HREE), whereas Opx_3_ in Type B peridotite is depleted in HREE and shows a positive Ti anomaly. Opx_4_ is moderately enriched in HREE. Panels (c,d) show PM-normalized trace element patterns of clinopyroxene (Cpx_2/3_) and amphibole (Amp_3_) in Type A and B peridotites. They show positive Pb anomalies and negative anomalies in high field strength elements. The trace element pattern of Amp in the Nonsberg peridotite^6^ is shown for comparison. Panel (e) shows PM-normalized trace element patterns of Garnet (Grt_2/3_) in Type A peridotites, in which pyrope (Prp)-poor Grts (~67 mol.%) are poor in HREE and Ti. Panel (f) shows trace element patterns of Prp-rich Grts (~70 mol.%) in Type B peridotites, which are rich in HREE and Ti.

Garnet-poor (~2 vol.%) Type A peridotite contains centimetre-size crystals of orthopyroxene and garnet that formed during Stages 1 and 2. Type A peridotite contains centimetre-size orthopyroxene porphyroclasts^7^ (Fig. 2c), the cores of which (Opx_1_, where the subscript represents the crystallization stage) contain 3 wt.% Al_2_O_3_, consistent with equilibration under high-temperature Stage 1 conditions (Fig. 1a). Large garnet crystals, which crystallized at Stage 2 (i.e., Grt_2_), contain abundant inclusions of chromite (Chr_1_) (Fig. 2d). Chr_1_ grains potentially retain a record of temperatures during Stage 1. Here we used Zn-in-chromite thermometry^17^ to constrain Stage 1 temperatures. The Zn concentrations in Chr_1_ in garnet (250–950 ppm) are systematically lower than those in matrix Chr_3_ (1320–5500 ppm) (Fig. 1b). In particular, Chr_1_ inclusions in large Grt_2_ grains preserve low Zn concentrations (250–400 ppm). Calculated crystallization temperatures for such Zn-poor Chr_1_ crystals are >1180 °C (Fig. 1b). At this temperature, the 3 wt.% Al_2_O_3_ in orthopyroxene isopleth constrains pressure at 2.8–3.6 GPa (Fig. 1a). Opx_1_ shows enrichment in LILE (Rb ≈ 1 ppm, Ba ≈ 16 ppm; Fig. 3a; Supplementary Table S2), a pronounced depletion in Ti (Ti_N_ = 0.1, where Ti_N_ is the measured composition normalised against the primitive mantle value^18^), a fractionated REE pattern with a high La_N_/Yb_N_ value (≈10) and relatively flat LREE contents (La_N_/Sm_N_ ≈ 0.5) (Supplementary Fig. S1a). A negative Ti anomaly in orthopyroxene is observed only in clinopyroxene-free harzburgite^19^. This finding is consistent with the absence of clinopyroxene porphyroclast, which constrains Stage 1 temperatures to above the clinopyroxene-out curve in the hydrous peridotite system (>1120 °C^20^).

Garnets in Type A peridotites are relatively poor in pyrope (~67 mol.%, Supplementary Table S1) and Na (<5 ppm) relative to those in Type B peridotites, and exhibit a pronounced depletion in Ti (Ti_N_ < 0.1) (Supplementary Table S2) and sinusoidal REE patterns with low LREE, HREE (La_N_ ≈ 0.5, Yb_N_ ≈ 2) and high MREE (Nd_N_ ≈ 7) concentrations (Fig. 3e and Supplementary Fig. S1e). Cpx_2_ within embayments in Grt_2_ is depleted in Na_2_O (0.3 wt.%; Supplementary Table S1) and shows positive Pb anomalies, negative HFSE anomalies (Ti_N_ ≈ 0.05) (Fig. 3c), and fractionated REE pattern (La_N_/Yb_N_ > 3000) (Supplementary Fig. S1c). The application of garnet–clinopyroxene geothermobarometry^21–23^ yields 810 °C at 2 GPa (Fig. 1c), corresponding to Stage 2. The peridotite matrix consists of fine-grained (~0.1 mm) Ol_3_, Opx_3_, Cpx_3_, Ca-Amp_3_, Chr_3_, Phl_3_ and Apt_3_ that crystallized during Stage 3. Where garnet rims are in contact with Na_2_O-rich (~1 wt.%) Cpx_3_ (Supplementary Fig. S2a), they are relatively enriched in pyrope (~70 mol.%), Na (35 ppm), Ti (Ti_N_ > 0.2), and HREE (Yb_N_ ≈ 10) (Fig. 3e, Supplementary Fig. S1e), suggesting recrystallization during Stage 3. Amp_3_ contains a lot of Chr_3_ inclusions (Supplementary Fig. S2b), and it coexists with Cpx_3_ in the matrix (Supplementary Fig. S2c). The trace element compositions of both Amp_3_ and Cpx_3_ are characterised by positive Pb anomalies and a marked depletion in HFSE (Nb, Ta, Zr, Hf and Ti; Ti_N_ ~ 1 in Amp_3_ and 0.2 in Cpx_3_) (Fig. 3c), and are rich in LREE (La_N_ ≈ 30–50 in both Amp_3_ and Cpx_3_) (Supplementary Fig. S1c). High LILE and low HFSE contents in Amp_3_ are similar to those in amphibole from the Nonsberg peridotite^6^. Matrix Opx_3_ in Type A peridotite exhibits a convex-downward REE pattern characterised by a relative enrichment in HREE (Dy_N_/Yb_N_ ≈ 0.1–0.2; Yb_N_ ≈ 0.4–0.6).

Garnet-rich (~5 vol.%) Type B peridotites have an inequigranular texture in which large spheroidal garnet grains (5–30 mm in length) occur within a millimetre-sized matrix of Ol_3_ + Opx_3_ + Cpx_3_ + Chr_3_ + Phl_3_ + Amp_3_ that equilibrated during Stage 3^7^ (Fig. 2e). Grt_3_ grains are rich in pyrope (~70 mol.%), Na (65 ppm), Ti (Ti_N_ ≈ 0.6) (Fig. 3f), and HREE (Yb_N_ ≈ 10, La_N_/Yb_N_ ≈ 0.01; Supplementary Fig. S1f). Grt_3_ grains contain abundant Opx_3_ and Ol_3_, along with subordinate Cpx_3_ and Phl_3_ (Fig. 2e, Supplementary Fig. S2d). Grt_3_ contains rare chromite inclusions that have concave surfaces against their host, suggesting that Chr_1_ grains were consumed to grow Grt_3_^7^. Opx_3_ grains are relatively depleted in Al_2_O_3_ (1.5 wt.%^7^) and LILE (Rb, Ba < 0.03 ppm; Supplementary Table S2), and show variable enrichment in LREE (La_N_ ≈ 0.01–0.10). A positive Ti anomaly (Ti_N_ ≈ 0.4) and low HREE (Yb_N_ ≈ 0.03–0.06) contents in Opx_3_ (Fig. 3b) are consistent with their crystallization in equilibrium with the garnet lherzolite assemblage. Cpx_3_ grains show high contents of Na_2_O (~1 wt.%), a positive Pb anomaly, negative anomalies in HFSE (Ti_N_ ≈ 0.4) (Fig. 3d) and fractionated REE patterns (Supplementary Fig. S1d; La_N_/Yb_N_ > 70). Application of garnet–clinopyroxene geothermobarometers^21–23^ to Grt_3_–Cpx_3(in Grt)_ pairs yields conditions of ~3 GPa at 950–1000 °C (Fig. 1d). Garnet crystals were transformed into kelyphite during Stage 4 (Fig. 2f), in which the kelyphite consists of Opx_4_, Cpx_4_, and spinel (Spl_4_) with minor Ca-Amp_4_^7^. Opx_4_ that formed at this stage shows extremely high HREE contents (Yb_N_ > 1; Fig. 3b and Supplementary Fig. S1b), presumably inherited from the breakdown of garnet.

## Reconstruction of melt compositions using multiphase solid inclusions

Multiphase solid inclusions (MSIs) have been widely observed in high-pressure minerals within UHP metamorphic rocks and orogenic garnet peridotites, and are interpreted as the crystallization products of dense, solute-rich supercritical fluids or melts^11,24^. Many studies have been undertaken on MSIs in order to reconstruct the original chemical composition of supercritical fluids or melts formed in subduction zones^11,13,24^. The Plešovice garnet peridotite preserves fossilized melts as MSIs that consist of volatile-rich minerals^7,13^. Based on rare accessory minerals, including priderite and burbankite, parental melts to the MSIs are speculated to have been kimberlite- or carbonatite-like^13^, although quantitative estimates of melt compositions are lacking.

**Figure 4:**
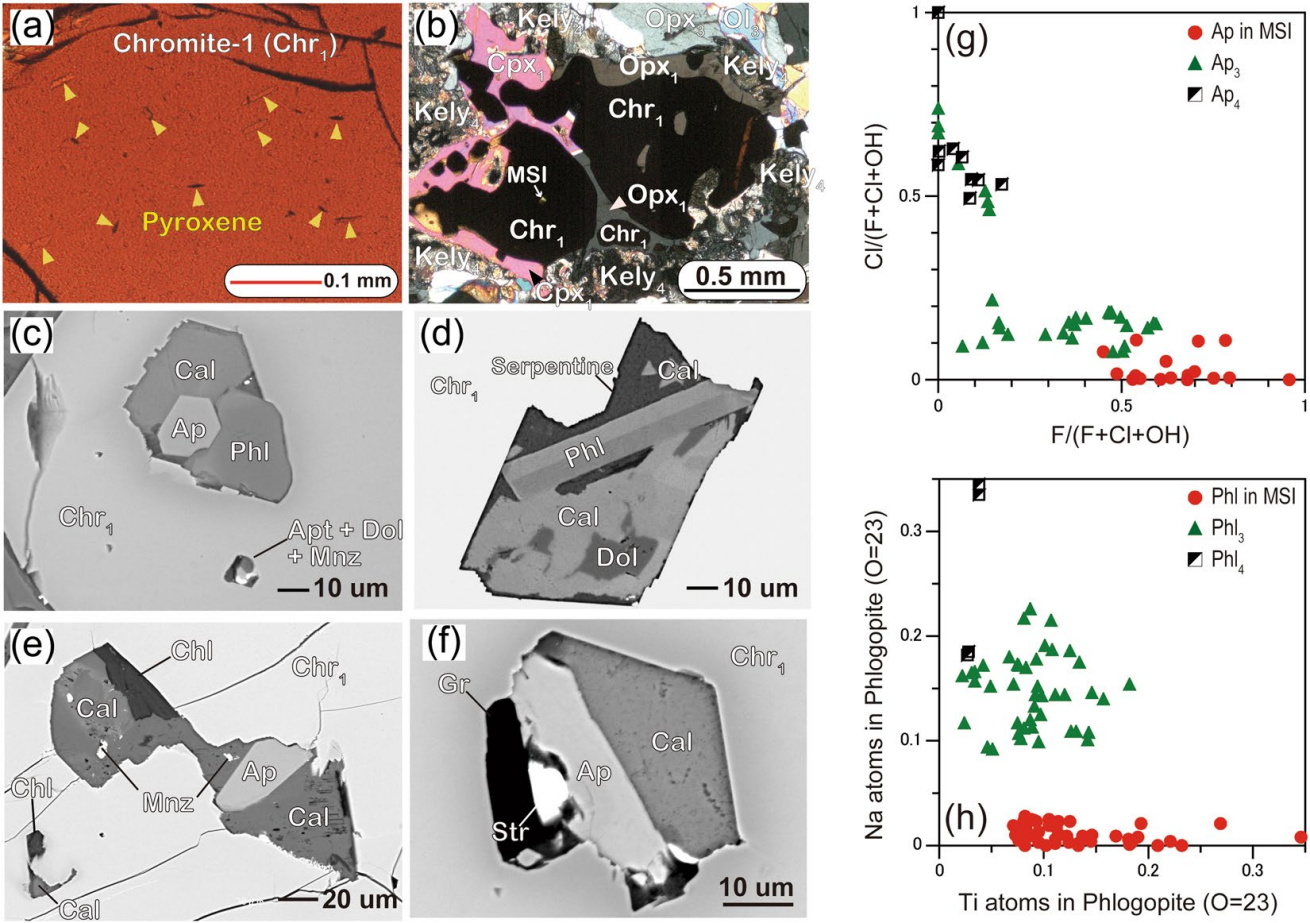
Panel (a) is a photomicrograph of a chromite grain (Chr_1_) containing pyroxene lamellae, indicating the former presence of chromite (Chr_0_) that grew under ultrahigh-pressure (UHP) conditions in the diamond stability field during Stage 0^12^, plane polarized light. Subscript numbers represent crystallization stages of minerals. Panel (b) shows aggregates of chromite (Chr_1_) + orthopyroxene (Opx_1_) + clinopyroxene (Cpx_1_) formed from UHP Chr_0_ by a recrystallization under the Stage 1. They are surrounded by kelyphite (kely_4_) of Stage 4. The matrix consists mainly of Stage 3 olivine (Ol_3_) and orthopyroxene (Opx_3_), cross polarized light. Panels (c) and (d) show back-scattered electron (BSE) images of multiphase solid inclusions (MSIs) consisting of phlogopite (Phl), calcite (Cal), dolomite (Dol) and apatite (Ap). Panels (e) and (f) show BSE images of MSIs consisting of Cal, Dol and Ap. Mnz = monazite, Str = strontianite, Gr = graphite, Chl = chlorite. Panel (g) is a diagram of F/(F + Cl + OH) versus Cl/(F + Cl + OH) in Ap occurring as MSIs in Chr_1_, matrix Ap_3_ and Ap_4_-proximal kelyphite. Panel (h) is a diagram of Ti versus Na contents in Phl from MSIs within Chr_1_, matrix Phl_3_ and Phl_4_-proximal kelyphite.

All observed Plešovice MSIs are within Chr_1_ grains enclosed in Grt_2_/Grt_3_ crystals. Large grains (>3 mm in length) of Chr_1_ contain exsolution lamellae of diopside and enstatite^12^ (Fig. 4a), suggesting that chromite contained pyroxene components during Stage 0^12^ (i.e. Chr_0_). However, we found no MSIs in these larger Chr_1_ grains. Smaller Chr_0_ grains recrystallized into exsolution-free chromite (Chr_1_) + diopside (Cpx_1_) + enstatite (Opx_1_) (Fig. 4b), and most MSIs occur in these exsolution-free Chr_1_ grains. Chr_1_ has a relatively high Cr# [molar Cr/(Cr + Al) = 0.57–0.64] but is deficient in Zn (as low as ~350 ppm; Fig. 1b) and equilibrated during Stage 1 (Fig. 1a). MSIs show negative crystal shapes and consist mainly of hydrous aluminosilicate minerals, phosphates and carbonates (Fig. 4c–f). Single-phase phlogopite inclusions also occur within Chr_1_. The presence of garnet in some MSIs (Supplementary Fig. S2e) suggests that the original melts were stable in the garnet lherzolite facies. For more information on variations in the minerals in MSIs, see the Supplementary Methods (Variations in multiphase solid inclusions and the cut effect on volume estimates and Supplementary Figs S3 and S4).

We examined 37 polished thin sections by electron microscopy and found 243 inclusions that are free of severe secondary alteration (Supplementary Table S3). These inclusions occur mainly within Type A peridotites (187), but some occur within Type B peridotites (56). The main carbonates in the MSIs are dolomite and calcite, with a dolomite/calcite volume ratio of 2.0 ± 0.6. Rarely, carbonates are magnesite, Ba–Ca–Mg carbonate, strontianite, and burbankite^13^. Other minerals identified in the MSIs include Nb-rich rutile, a Mn–Fe–Ti–W–Ta phase (possibly koragoite), Cr-priderite^13^, monazite, a U–Th oxide and various sulphide minerals including maucherite, orcelite, millerite, galena and pentandite (Supplementary Table S3). The occurrence of such a wide variety of minerals in the MSIs can be explained by use of the phase rule (see Supplementary Discussion – application of the phase rule to multiphase solid inclusions). Some MSIs contain chlorite, calcic amphibole, talc, serpentine, brucite, and diaspore. These MSIs are connected with the matrix via annealed fractures (Supplementary Fig. S2f). We did not use such altered MSIs for the reconstruction of melts.

**Figure 5:**
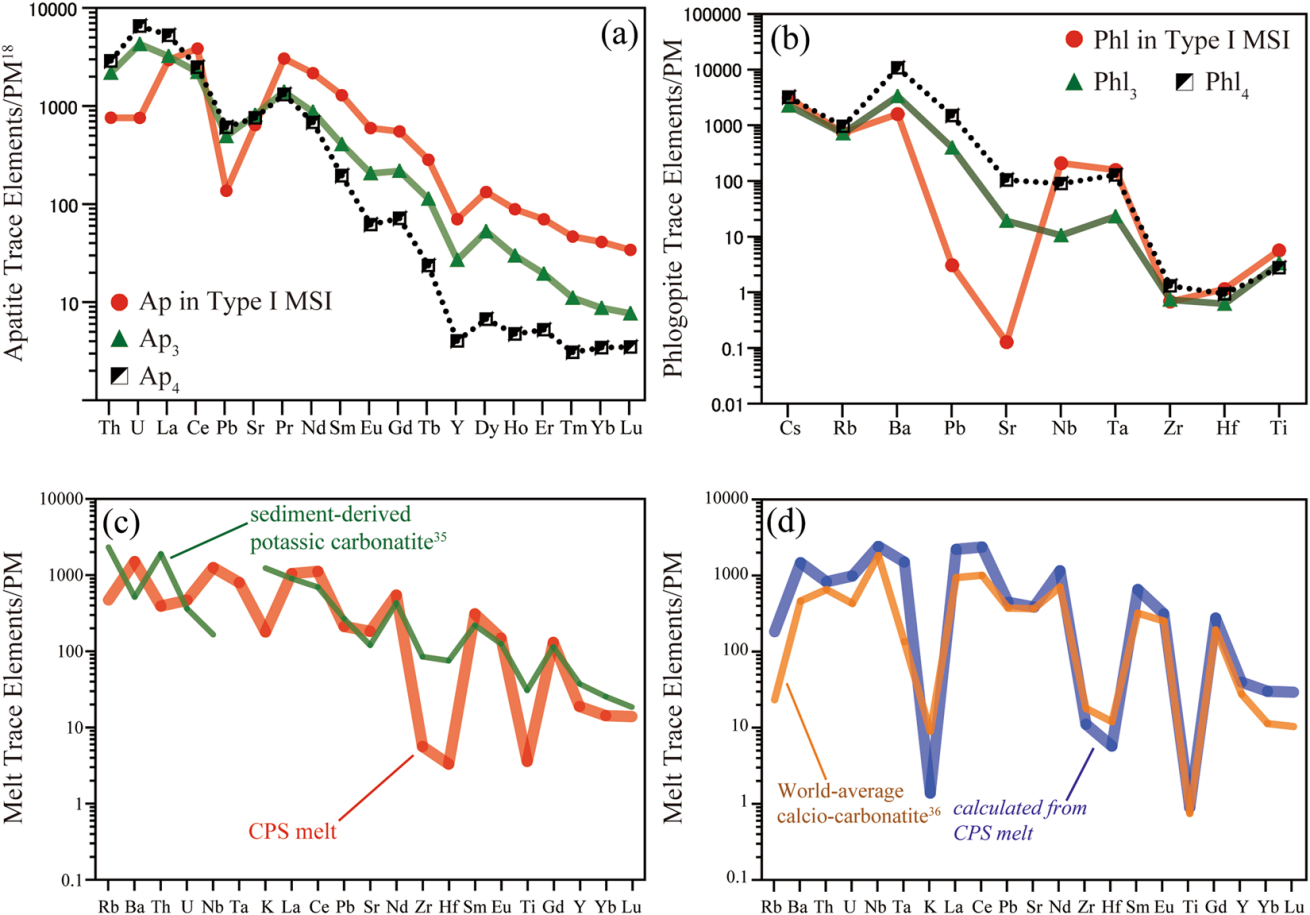
Panel (a) shows a trace element patterns of apatites (Aps), in which the subscripts indicate the crystallization stage, normalized to primitive mantle values^18^. MSI = multiphase solid inclusion. Panel (b) shows trace element patterns of phlogopites. Panel (c) displays a trace element pattern for a carbonated potassic silicate (CPS) melt calculated using trace elements in Ap and Phl within MSIs (Supplementary Table S2) in combination with appropriate partitioning coefficients for these minerals and carbonatite melts^33,34^. For comparison, the trace element patterns of a sediment-derived potassic carbonatite^35^ are shown. Panel (d) shows a trace element pattern for a carbonatite that formed from the CPS melt by the precipitation of phlogopite. A trace element pattern for global average calcio-carbonatite^36^ is shown for comparison.

Crystals of apatites and phlogopites are ubiquitous within both the MSIs and the matrix, and their chemical compositions correlate with textures, for both major elements (Fig. 4g,h; Supplementary Table S1) and trace elements (Fig. 5a,b; Supplementary Table S2). Apatite grains in MSIs are rich in F {*X*_F_ = F/(F + Cl + OH) > 0.45, *X*_Cl_ = Cl/(F + Cl + OH) < 0.1} and Ce relative to U (Ce_N_/U_N_ > 3), and are clearly distinguished from apatites in the surrounding kelyphite (X_F  _< 0.1, X_Cl_ > 0.5; Ce_N_/U_N  _< 1) (Figs 4g, 5a). Apatites in the MSIs show high REE contents, with fractionated REE patterns (Ce_N_/Yb_N_ ≈ 100) (Fig. 5a). Phlogopite grains in MSIs are strongly deficient in Na ( < 0.03 cations per 23 oxygens) and depleted in Pb and Sr, whereas phlogopite grains in both the matrix and proximal kelyphite are rich in Na (>0.1 cations per 23 oxygens), Pb and Sr (Figs 4h, 5b). The observed large compositional gaps between apatites and phlogopites in MSIs, compared with those in the matrix, suggest that the MSIs formed under different physical and chemical conditions to the matrix minerals.

The bulk chemical compositions of MSIs in Type A and Type B peridotites have been reconstructed using the mineral proportions (vol.% in Supplementary Table S3) and mineral compositions (Supplementary Table S1). In spite of the variable proportions of constituent minerals in each of the MSIs due to the cut effect (Supplementary Figs. S3a and S4c), their average volumes converge to constant values (Supplementary Fig. S3b) after the accumulation of more than 80 data points. This verifies our assumption that the chemical compositions of the MSIs are scattered around a meaningful average.

**Figure 6:**
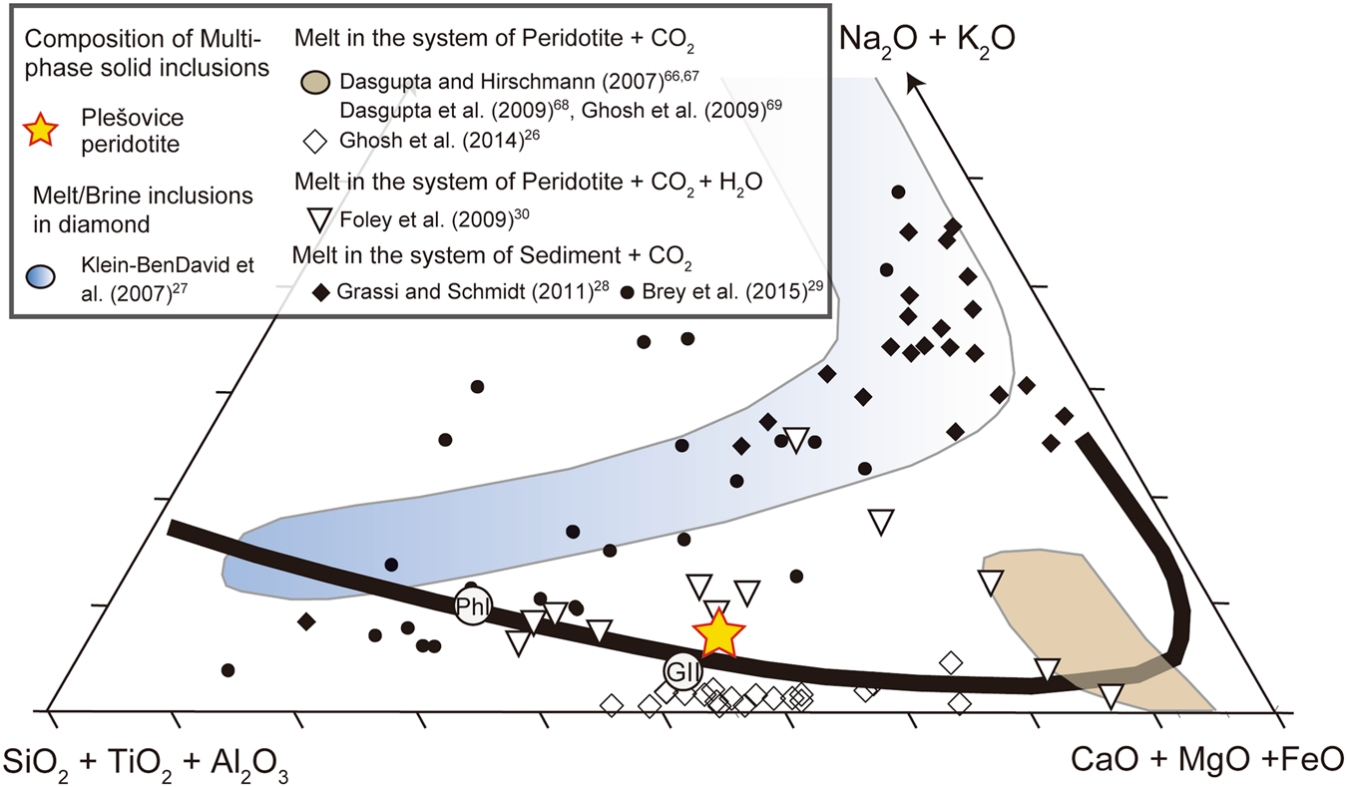
This panel shows a summary of carbonated silicate melts preserved as multiphase solid inclusions (MSIs) in the Plešovice peridotite in comparison with those produced by experiments^26–30,66–69^. Ternary (wt.%) diagram projected from CO_2_ and H_2_O. The average compositions of group II kimberlites^25^ (GII) and of phlogopite (Phl) are shown. The thick curve represents the solvus for a silicate–carbonatite liquid at 1100 °C and 3.7–5.0 GPa^28^. The mean value of carbonated melt reconstructed from MSIs in the Plešovice peridotite is represented as a yellow star which is close to the composition of GII kimberlites.

The results are plotted on a ternary diagram (Fig. 6) of (SiO_2_ + TiO_2_ + Al_2_O_3_), (CaO + FeO + MgO), and (Na_2_O + K_2_O) wt.% projected from CO_2_ and H_2_O. The parental melt composition is K_2_O = 5.2 wt.%, CaO = 17 wt.%, MgO = 18 wt.%, CO_2_ = 22 wt.%, and SiO_2_ = 20 wt.%, giving an average K_2_O/Na_2_O ratio of ~120 (Supplementary Table S1). This chemical composition is similar to that of Group II kimberlites^25^ and suggests that the carbonated potassic silicate melts (hereafter referred to as CPS) formed in equilibrium with a garnet-harzburgite residue at temperatures above the carbonated peridotite solidus (i.e. *T* > 1200 °C^25^). Although the alkali contents in the CPS melts are much higher than those of melts in the peridotite–CO_2_ system^26^, the alkali contents are much lower than those of melt inclusions within diamond^27^ and sediment-derived carbonatites^28,29^. CPS melts with K_2_O contents of ~5 wt.%, similar to those measured in our samples, have been produced experimentally as partial melts in the peridotite–CO_2_ system doped with 1.5 wt.% K_2_O^30^ (Fig. 6). Therefore, a source for the CPS melt may have been mantle peridotite that was metasomatized by potassic melts. As shown in Fig. 1, the CPS melt could have been stable within the Plešovice peridotite under the conditions of Stage 1 (~1300 °C and 3 GPa), under which chromite is stable in garnet harzburgite^31^ (Fig. 1). Upon cooling, the rock would have crossed reaction curve (1) at ~1220 °C^32^, at which point the melt would have changed to a calcio-carbonatite. We hypothesize that CPS melt crystallizes to form roughly equal proportions of carbonatite melt and phlogopite, resulting in production of a melt with the following composition: MgO = 12 wt.%, CaO = 35 wt.%, CO_2_ = 47 wt.%, and SiO_2_ < 1 wt.% (Supplementary Table S1).

The trace element compositions of the CPS melts were calculated using the compositions of apatite and phlogopite (Supplementary Table S2) using the following relation: *C*_i_ (melt) = *D*_i_ (melt/crystal) × *C*_i_ (crystal), where *D*_i_ represents the partition coefficient of element *i*^33,34^ (see Methods). The calculated trace element pattern for the CPS melt is similar to those of sediment-derived carbonatites^35^ (Fig. 5c), showing significant enrichment in LILE such as Rb, Ba, Th, and U, and light REE. However, the quantity of HFSE in the CPS melt is much lower than in sediment-derived carbonatites^35^ (Fig. 5c). Fractional crystallization of phlogopite transformed the CPS melt to a carbonatite, whose trace element pattern is represented by the blue line in Fig. 5d. This composition closely resembles that of average calcio-carbonatite lavas worldwide^36^ (Fig. 5d). Subsequently, the calcio-carbonatite was likely transformed into a carbonatitic fluid through the reaction of carbonatite and enstatite to diopside, forsterite and CO_2_^37^.

## Equilibrium modelling of REE between minerals and melts

**Figure 7:**
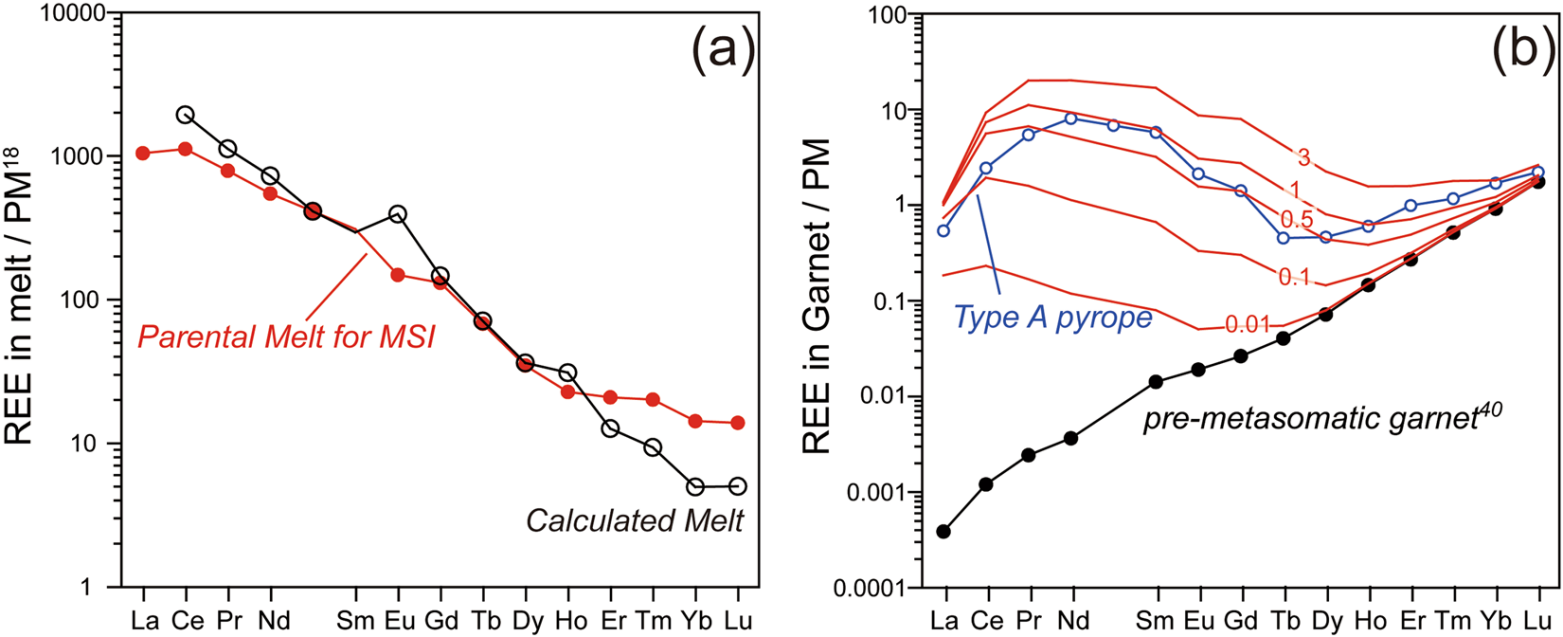
Panel (a) shows a calculated REE pattern of a carbonated melt in equilibrium with the core of an orthopyroxene porphyroclast, normalized to primitive mantle (PM) values^18^. The calculated REE pattern of the melt agrees well with that of the reconstructed REE pattern for the MSIs. Panel (b) shows a result of REE modelling of a reaction between pre-metasomatic garnet^40^ and a calculated carbonatite melt. The red-coloured curves are calculated patterns for varying volumes of carbonatite melt that reacted with garnet. Garnet–melt partition coefficients are after ref.^39^. Only 0.5–1.0% carbonatite melt relative to garnet is needed to generate the observed sinuous REE pattern in Type A peridotite.

Here we aim to decipher the metasomatic signatures recorded in silicate minerals that were stable under the conditions of Stages 1 and 2, and we examined whether the CPS melt and carbonatite were in equilibrium with the residual silicate minerals. Firstly, orthopyroxene and olivine in Type A peridotite have high Mg# [Mg/(Mg + Fe^2+^)] (Mg#_Opx_ ≈ 92 in Table S1; Mg#_Ol_ ≈ 91.5^7^). Due to their relative insensitivity to changes in *T* and *P*, the Mg# of orthopyroxene and olivine serve as indicators of melt depletion^19^. At ~3 GPa, an olivine Mg# of 91.5 equates broadly to the point of complete clinopyroxene consumption at ~25% partial melting^19,38^. Modelling that considers batch melting has shown that orthopyroxene in a residual peridotite acquires a REE pattern with a high HREE/LREE ratio^19^. Therefore, the observed high LREE content in Opx_1_ cannot be formed as a result of partial melting events, but it requires the metasomatic addition of LREE after partial melting. Here we examine the hypothesis that a CPS melt coexisted with Opx_1_ during Stage 1. To test this hypothesis, we calculate REE patterns of a hypothetical melt that coexisted with Opx_1_, utilizing partitioning coefficients determined by an experiment at 1300 °C and 6 GPa (Run#: M355^39^). As shown in Fig. 7a, the calculated REE pattern of melt coexisting with Opx_1_ matches fairly well with that of the CPS melt. This suggests that the CPS melt was a metasomatic agent during Stage 1.

After Stage 1, the CPS melt crystallized phlogopite, was transformed to carbonatite, and then may have been converted into a carbonaceous fluid during cooling to Stage 2. It has been shown that carbonaceous fluids derived from carbonatite have similar trace element compositions to the source carbonatite^40^. Both are strongly enriched in incompatible trace elements and have the potential to significantly alter the trace element signatures of Stage 2 minerals. Grt_2_ in Type A peridotite shows sinuous REE patterns (Fig. 7b) that may have been the product of metasomatism by LREE-rich metasomatic agents^41^. Here we attempt to reproduce the sinuous REE patterns in Grt_2_ by modelling interaction between a pre-metasomatic garnet^40^ and the calculated composition of the carbonatite melt. Firstly, we assume various proportions of melt (0.01, 0.1, 0.5, 2, and 5 wt.%) relative to the mass of pre-metasomatic garnet^40^. To simulate the reaction between carbonatite melt and garnet, we solved following two equations simultaneously, (1) *C*_REE_ (garnet) + *p* × *C*_REE_ (carbonatite) = constant (mass-balance equation), and (2) *C*_REE_ (garnet)/*C*_REE_ (carbonatite) = *D*_REE_ (garnet/carbonatite)^39^, where *p* represents proportion of melt. This modelling shows that only 0.5–1.0 wt.% melt relative to garnet can produce the observed sinuous REE pattern observed in Grt_2_ grains of Type A peridotites (Fig. 7b). As Type A peridotite contains 2 vol.% garnet, the required melt fraction is only about 0.02–0.04 vol.% (the densities of pyrope and carbonatite are assumed to be 3.7 and 2.2 gcm^–3^, respectively).

## Discussion

We have reconstructed the major and trace element composition of carbonated potassic silicate (CPS) melt inferred to have been present in the mantle wedge during Stage 1 (~1300 °C at 3 GPa). This melt could have been sourced from sedimentary rocks subducted to depths of > 250 km^28^. Infiltration of the sediment-derived carbonatite (i.e. K_2_O > 27 wt.%, CaO = 17 wt.%, CO_2_ = 38 wt.%, SiO_2_ = 0.3 wt.% in run am-5B^28^) into the mantle produces a phlogopite-bearing peridotite. Due to the highly reduced conditions in the deep mantle wedge, the carbonatite may have recrystallized to diamond through the reduction of carbon dioxide during Stage 0^12,42^ (Fig. 1). Subsequent convective upwelling of the volatile-rich peridotite induced melting beneath the overriding lithosphere by oxidation^42^, producing a CPS melt during Stage 1 (Fig. 1). The low abundances of HFSE in the CPS melts can be explained by the persistence of ilmenite in a residual peridotite to temperatures 100–120 °C above the solidus^30^.

It has been proposed that the *P–T* paths of mantle wedge peridotites are controlled by corner flow^43^, which can be envisaged as two types (Supplementary Fig. S5). Models of slab-induced flow in the mantle wedge show that the flow lines are sub-horizontal and directed towards the slab in the uppermost part of the mantle wedge^44^, consistent with the initial *P–T* paths observed for the Nonsberg^43^ and Higashi-Akaishi peridotites^45,46^. However, this model cannot account for initial upward flow, such as that inferred for the Plešovice and Zhimafang peridotites^8^. The volatile contents in the mantle wedge potentially control the style of corner flow^5^, as well as directly influencing the chemical compositions of mantle wedge melts. Mantle wedge peridotites originating in the spinel lherzolite facies record magmatic activity producing high-temperature silicate melts^6,45,46^, whereas those originating in the garnet lherzolite facies preserve volatile-rich melts as MSIs. In the Zhimafang peridotite, an alkali-rich hydrous silicate melt stable at ~5 GPa has been identified based on the study of MSIs (consisting of calcic amphibole, chlorite, phlogopite, and talc) enclosed within primary garnet^11^. Thus, we propose that volatile-rich partial melts can create upward flow in the mantle wedge.

When the CPS melt cooled below 1220 °C it started to crystallise phlogopite. Thus, phlogopite crystallization should have taken place near the base of the lithosphere or a short distance above the thermal boundary layer (~1300–1400 °C)^47^. Recent high-resolution seismic studies have demonstrated a substantial reduction in shear wave velocities (*V*_S_) at depths of 60–150 km in the cratonic lithosphere^48,49^, in the oceanic lithosphere–asthenosphere boundary (LAB)^50,51^ and in the mantle wedge^52^. Several hypotheses have been proposed to explain this reduction in *V*_S_: 1) the presence of partial melt^50,51^ 2) elastically accommodated grain boundary sliding^53^ and 3) the presence of hydrous minerals produced through metasomatism^48,49^. Phlogopite is seismically slow and can cause a reduction in *V*_S_. For example, the formation of 5–7 vol.% phlogopite in the lithosphere can reduce *V*_S_ by up to 6%^49^. This velocity reduction matches values observed at the oceanic LAB (a 7–9% reduction^50,54^) and can explain modest velocity reductions observed in the cratonic lithosphere (a 2%–24% reduction^48,49^).

Recently petrologists have proposed that physical mixing of subducted sediment and mantle wedge peridotites may result in the formation of volatile-rich hybrid rocks in low-temperature subduction zones^55^. Such hybrid rocks could create low-*V*_S_ regions in the subduction mélange. In warmer subduction/collision zones, subducted sediment would produce potassic carbonatites^28^. Interaction between potassic carbonatites and mantle peridotites results in the creation of phlogopite-bearing carbonated peridotite^5^ or a phlogopite-rich metasomatic layer (i.e. glimmerite^56^). In both cases, blebs of hybrid rocks or phlogopite-bearing carbonated peridotite/glimmerite will rise buoyantly from the surface of the subducting slab into the sub-arc mantle, producing low seismic anomalies along their ascent pathways^55^. Finally, these diapirs would melt to produce potassic melts. Melting of phlogopite in the mantle wedge generates ultrapotassic magmas with high K_2_O and MgO (>3 wt.%)^57^, such as those observed in the Bohemian massif. Orogenic garnet peridotite in the Bohemian Massif (Austria) contains glimmerite veins^58^ as well as CPS melts. Both are key features that give rise to orogenic ultrapotassic magmatism.

In this study, we constrained the composition of mantle wedge melts. High contents of LILE, LREE and carbon in the CPS melts were likely derived from subducted carbonate-rich sediments^28,56^. Although the presence of this type of potassium- and volatile-enriched melt in the asthenosphere has been speculated to exist^59,60^, and is sometimes referred to as “failed kimberlite”^25^, this study is the first to report a natural example from mantle wedge samples. Such CPS melts may be ubiquitous in the deep mantle wedge, but they are rarely present in sufficient quantity to reach the Earth’s surface. Due to small melt fractions, the CPS melts are expected to have differentiated to carbonaceous fluid within the lithosphere, with carbon dioxide returned to the surface through arc volcanism^1^.

## Methods

Major element analyses were performed using an EPMA (JEOL JXA-8900L at the University of Tokyo) and a FE-EPMA (JEOL JSM-8350F, at the University of Tokyo). Most silicate minerals, carbonates, phlogopite, and phosphates were analysed by EPMA. The analytical conditions were 15 kV, a surface beam current of 12 nA [1–3 µm beam diameter (b.d.)] for silicates and phosphates, and 3 nA (scanning mode with magnification of 20,000) for carbonate with a ZAF correction. The sulphides, priderite, burbankite, Ba-rich carbonates, and monazite were analysed by FE-EPMA. Analytical conditions were 15 kV and a surface beam current of 6 nA (3 μm b.d.) for priderite, 15 kV and 12 nA (1 um b.d.) for sulphides, and 25 kV and 20 nA (10 μm beam diameter) for Ba-rich carbonates, burbankite, and monazite. Oxides, silicate minerals, and natural and synthetic carbonates with known compositions were used as analytical standards.

Trace element analyses were performed on an iCAP Q inductively coupled plasma mass spectrometer (Thermo Fisher Scientific) attached to an LSX-213 G2 + Nd:YAG laser ablation system (Cetac Technologies) at the University of Tokyo. Each analysis consisted of a 25-sec baseline measurement followed by a ~35-sec measurement with laser ablation sampling. The data were obtained from 15–25 µm ablation pits with a laser repetition rate of 4–8 Hz. An external calibration was made against NIST 611 SRM, and an internal standard calibration was applied using the Ca (apatite and garnet), Mn (chromite and orthopyroxene) and K (phlogopite) concentrations determined by EPMA.

Trace element concentrations in melts were calculated using those in apatite and phlogopite in multiphase solid inclusions (MSIs). According to the phase diagram and crystal shapes, the first mineral to crystallize from parental melt is phlogopite followed by apatite. The phlogopite crystallization reaction is as follows: Melt → 0.474 residual melt-1 + 0.526 phlogopite (wt.% basis). The concentrations of Na, Rb, Cs, Ba, Ta, Nb, Hf, Zr and Ni in a residual melt-1 were calculated using those in phlogopite grains in conjunction with trace element partitioning coefficients between phlogopite and carbonatite^34^. The apatite crystallization reaction is as follows: Melt → 0.442 residual melt-2 + 0.526 phlogopite + 0.032 apatite. Some trace elements (Pb, Th, U, and REE) in the residual melt (melt-2) were estimated using those in the apatite grains in combination with trace element partitioning coefficients between apatite and carbonatite^33^.

Partitioning coefficients of Rb, Ba, Nb, and Zr between phlogopite and carbonatite are from ref.^34^, and those of Cs and Ta are from ref.^61^. The partitioning coefficients of Hf was assumed to be 0.19^62^, which agrees with the average value in the literature^63^. In order to reduce the error of partitioning coefficients between apatite and carbonatite^33^, we calculated the standard error by dividing σ by the square root of the number of data (n = 60) (i.e. σ/√n)^33^.

The calibration of Zn in chromite geothermometer of ref.^17^ {*T* = *h* (Zn ppm)} adopted the temperature based on the Ni-in-garnet geothermometer of ref.^17^ {*T* = *f* (Ni ppm)}, which has been shown to be inconsistent with the calibration proposed by ref.^64^ {*T* = *g* (Ni ppm)}. Therefore, we corrected Zn-in-chromite thermometer as follows: *T* = *g* {*f* ^−1^ {*h* (Zn ppm)}} to ensure that it is consistent with the calibration proposed by ref.^64^.

## Electronic supplementary material

Supplementary Information
