# Author Correction: Fossilized Melts in Mantle Wedge Peridotites

**DOI:** 10.1038/s41598-020-59545-8

**Published:** 2020-02-12

**Authors:** Kosuke Naemura, Takao Hirajima, Martin Svojtka, Ichiko Shimizu, Tsuyosi Iizuka

**Affiliations:** 1 Nagoya University Museum, Furo-cho, Chikusa-ku, Nagoya 464-8601 Japan; 2 Department of Earth and Planetary Science, Graduate School of Science, The University of Tokyo, 7-3-1 Hongo, Bunkyo-ku, Tokyo 113-0033 Japan; 3 Division of Earth and Planetary Sciences, Graduate School of Science, Kyoto University, Kitashirakawa Oiwake-cho, Sakyo-ku, Kyoto 606-8502 Japan; 4 Institute of Geology of the Czech Academy of Sciences, Rozvojová 269, 165 00 Praha, Lysolaje Czech Republic

Correction to: *Scientific Reports* 10.1038/s41598-018-28264-6, published online 04 July 2018

In the Supplementary Information file originally published with this Article, there were errors in the affiliations 2,3 and 4, the subheading of the Supplementary Methods section and in the references in the legend for Supplementary Figure S5. These errors have been corrected in the Supplementary Information that now accompanies the Article.

