# Supplementary material for: Fossilized Melts in Mantle Wedge Peridotites
Source: Sci Rep. 2018 Jul 4;8:10116. doi: 10.1038/s41598-018-28264-6 (PMC6031665; doi:10.1038/s41598-018-28264-6)
Supplement: Supplementary file 1 — Supplementary Information [file 41598_2018_28264_MOESM1_ESM.pdf]

Supplementary Information for

Fossilized Melts in Mantle Wedge Peridotites

*Kosuke Naemura[1,2], Takao Hirajima[3], Martin Svojtka[4],
Ichiko Shimizu[2,3], and Tsuyosi Iizuka[2]

[1] *Nagoya University Museum, Furo-cho, Chikusa-ku, Nagoya, 464-8601, Japan.*

[2] *Department of Earth and Planetary Science, Graduate School of Science, The University of Tokyo, 7-3-1 Hongo, Bunkyo-ku, Tokyo, 113-0033, Japan.*

[3] *Division of Earth and Planetary Sciences, Graduate School of Science, Kyoto University, Kitashirakawa Oiwake-cho, Sakyo-ku, Kyoto, 606-8502, Japan.*

[4] *Institute of Geology of the Czech Academy of Sciences, Rozvojová 269, 165 00, Praha - Lysolaje, Czech Republic.*

* naemura@num.nagoya-u.ac.jp

Contents of this file

1. **Supplementary Figure S1:** REE element patterns of minerals in the Plešovice peridotite,
   Supplementary Figure S2: Photos of microtextures of the Plešovice peridotite.
2. **Supplementary Methods** – Variation of multiphase solid inclusions and the cut effect on volume estimates including ***Supplementary Figures S3 and S4***.
3. **Supplementary Discussion** – application of the phase rule to multiphase solid inclusions
4. **Supplementary Figure S5** which aims to compare pressure temperature paths of mantle wedge peridotites from high-pressure metamorphic terranes.
5. **Supplementary Tables S1, S2**: Mineral and melt compositions
   Supplementary Table S3: Volume percent of minerals in multiphase solid inclusions.

INTRODUCTION

The Supporting Information provides Supplementary Figs S1–S2, a Supplementary Methods – detail explanation about variation of minerals in multiphase solid inclusions and the cut effect on volume estimates with Supplementary Figs S3–S4, a Supplementary Discussion regarding the application of the phase rule to multiphase solid inclusions, and Supplementary Fig S5 in order to compare pressure-temperature path of mantle wedge peridotites from high pressure metamorphic terrane, and Supplementary Tables S1–S3.

1. Supplementary Figures S1 & S2

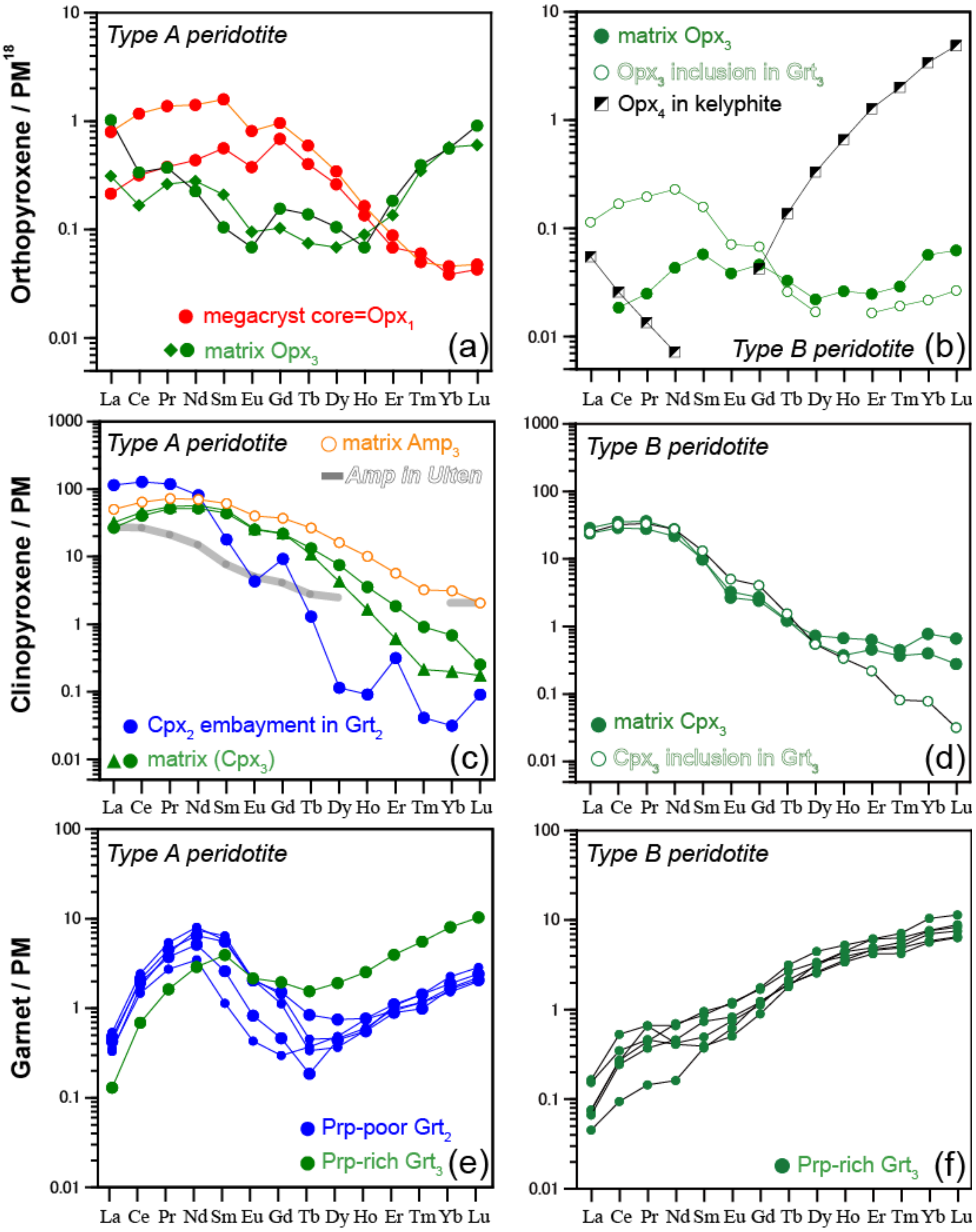

Supplementary Figure S1. Panels (a) and (b) show REE patterns of orthopyroxene (Opx) grains normalized by primitive mantle values[18], with subscript numbers denoting crystallization stages. Opx_1 is enriched in light REE (LREE) and is depleted in heavy REE (HREE). REE pattern of Opx_3 in Type A peridotite show a downward convex pattern with an increase in HREE, whereas those in Type B one show depletion in HREE. Opx_4 is rich in HREE. Panels (c) and (d) show a REE pattern of clinopyroxene grains ($Cpx_{2/3}$) and Amp_3 which are depleted in HREE. A REE pattern of Amp from the Ulten peridotite[6] is shown for comparison. Panels (e) and (f) show Pyrope (Prp)-poor garnet crystals (Grt_2) in Type A peridotites, showing sinusoidal REE patterns with low contents in both LREE and HREE, whereas Prp-rich garnet crystals (Grt_3) in Type B ones are enriched in HREE.

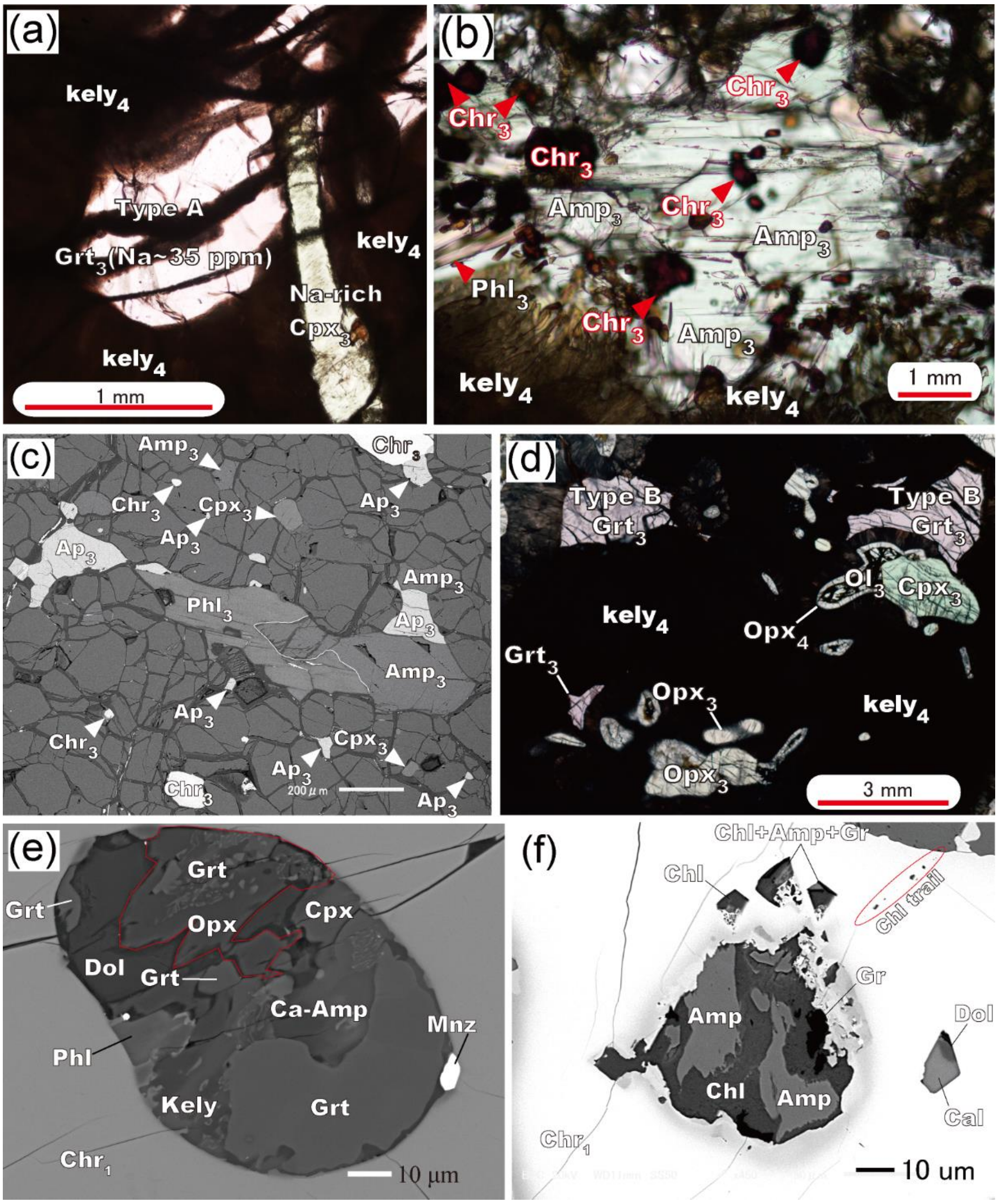

Supplementary Figure S2. Panel (a) is a close up view of garnet (Grt) rim in Type A peridotite, with stages are denoted by subscript numbers. The rim is rich in Na (~35 ppm) and is in contact with Na-rich (0.9 wt.%) clinopyroxene (Cpx_3). Panel (b) is a photomicrograph of a pargastic amphibole (Amp_3) with chromite inclusions (Chr_3). $Kely_4$ = kelyphite. Plane polarized light. Panel (c) is a back-scattered electron (BSE) image of apatite (Ap_3), phlogopite (Phl_3) and calcic amphibole (Amp_3) in the Type A peridotite. Panel (d) is a photomicrograph of Grt_3 that has been pervasively replaced by $Kely_4$ in Type B peridotite. The Grt_3 contains orthopyroxene (Opx_3), olivine (Ol_3) and a Cpx_3. Plane polarized light. Panel (e) shows a BSE image of a multiphase solid inclusion (MSI) consisting of Grt, Phl, Mnz, dolomite (Dol), Opx, Cpx, and Amp in a Chr_1 grain. A red curve is a boundary between Opx and Dol. Panel (f) is a MSI with a lot of chlorite (Chl), Amp and graphite (Gr). Chl-rich MSIs display irregular grain boundaries and are connected with the matrix through the trail of Chl.

2. Supplementary Methods – Variation of multiphase solid inclusions and the cut effect on volume estimates

As we are measuring data from arbitrary two-dimensional sections in order to estimate three-dimensional mineral volumes in multiphase solid inclusions (MSIs), it is worthwhile to consider the influence of the cut effect on volume estimates.

The constituent minerals in the MSIs are highly variable. A histogram of phlogopite modal abundance in the MSIs (Supplementary Fig.3a) allows the identification of three main populations: (1) MSIs consisting of phlogopite, carbonates and apatite (185 of 243 MSIs; Fig. 4c, d); (2) carbonate-rich MSIs containing <10 modal % phlogopite (22 of 243; Fig. 4e, f); and (3) phlogopite-rich inclusions with >90 modal% phlogopite (36 of 243). However, these populations are an artefact, a function of the two-dimensional section through the three dimensional MSI being examined, as will be shown in next page. In essence, within the studied MSIs, 51 vol.% of the MSIs is phlogopite, with the remainder being 39 vol.% carbonate, which gives a volume ratio of phlogopite/(phlogopite + carbonate) at ≅0.6.

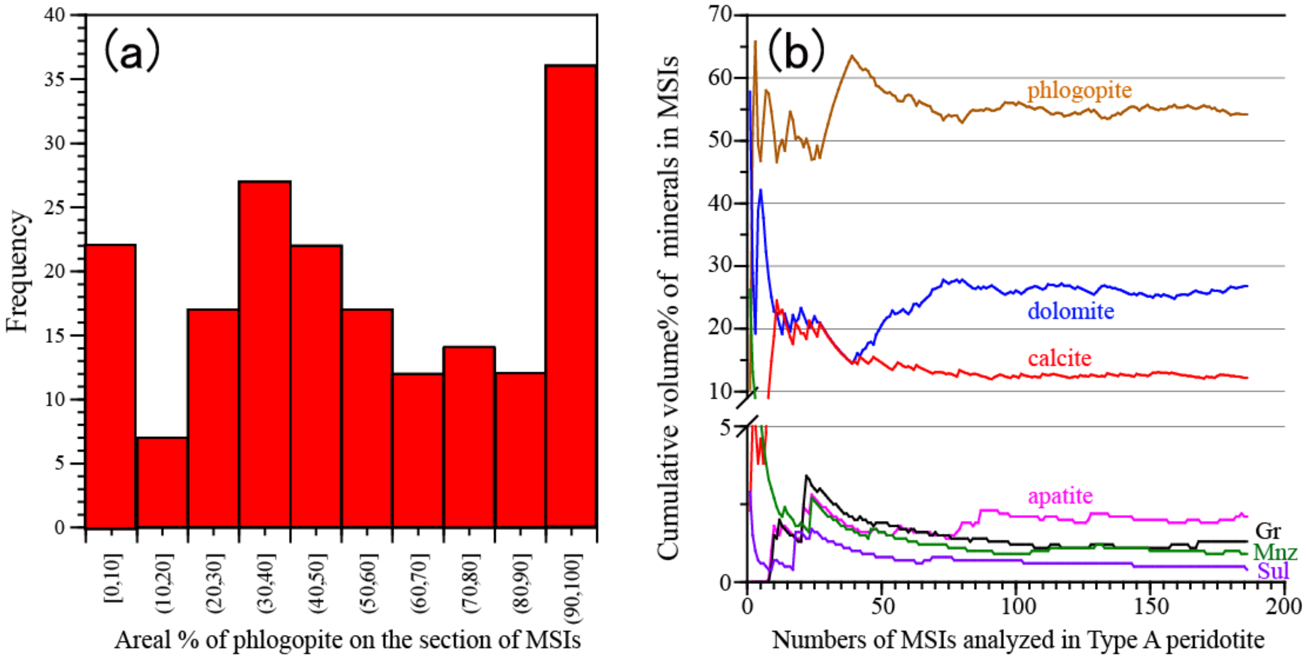

Supplementary Figure S3. Panel (a) shows a histogram of phlogopite volume % in MSIs from the Plešovice peridotites. The average of the frequency locates at ≅51 vol. %, with a significant spread of data. Two peaks, clustering at <10 vol. % phlogopite and >90 vol. %, reflect the situations that plane cut MSIs through either phlogopite or carbonate. Panel (b) shows cumulative proportions of minerals in MSIs from Type A peridotites. With the increase of data accumulation, mineral proportions converge to certain values. Gr=graphite, Mnz=monazite, and Sul=sulfides and sulphates.

Here we assume a cube-shaped MSI (1 cm^3) in which 60% of the volume is occupied by phlogopite and 40% by carbonate. We model cutting the MSI along a random plane (Supplementary Information Fig. S4a). Here, the orientations of planes are described by their orthogonal unit vectors. For example, the xy plane is described by the (0, 0, 1) vector. We generated an arbitrary unit vector to decide on a cutting plane. Firstly, we rotated the (0, 0, 1) vector around the x-axis by a given amount (a°), resulting in a vector of {0, sin (a), cos (a)}. This vector is again rotated clockwise around the y-axis by b° to give a vector of **u** = {sin (b) × cos (a), sin (a), cos (b) × sin (a)}. Finally, we use the cutting plane perpendicular to the vector **u** (Supplementary Fig. S4a). To simulate the random cutting of MSIs, we first produced 10 pairs of random integers (a, b) {$0 \leq a, b \leq 359$} that generate a vector **u** with random orientations. Then we translated the cutting plane along unit vector by 0.2 steps to intersect the MSI cube. Supplementary Figure S4b shows an example of this section. We measured the modal abundance of phlogopite in each section. After cutting the MSI 60 times in this way, we obtain a histogram of mode% phlogopite within the slices. This diagram shows the following.

(1) The average modal abundance of phlogopite in slices approaches the measured abundance of phlogopite (i.e., 60 vol.%) in three dimensions as the numbers of slices increases.
(2) We originally distinguished three types of inclusions: phlogopite–carbonate mixtures (Type I), carbonate-rich inclusions (Type II), and phlogopite-rich inclusions (Type III) which appear in the histogram of phlogopite vol.% (Supplementary Fig. S3a). Importantly, the cutting simulation produced several single-phase sections comprising either phlogopite or carbonate alone. Therefore, the aforementioned three types are artefacts. This view is supported by the similarity in the histograms of the modal proportion of phlogopite between those measured in natural samples (Supplementary Fig. S3a) and those modelled (Supplementary Fig. S4c).
(3) Notably, the significant spread of the peak at around 0.4–0.6 mode% phlogopite in the natural samples (Supplementary Fig. S3a) can be explained by the complex shapes of minerals in natural MSIs.

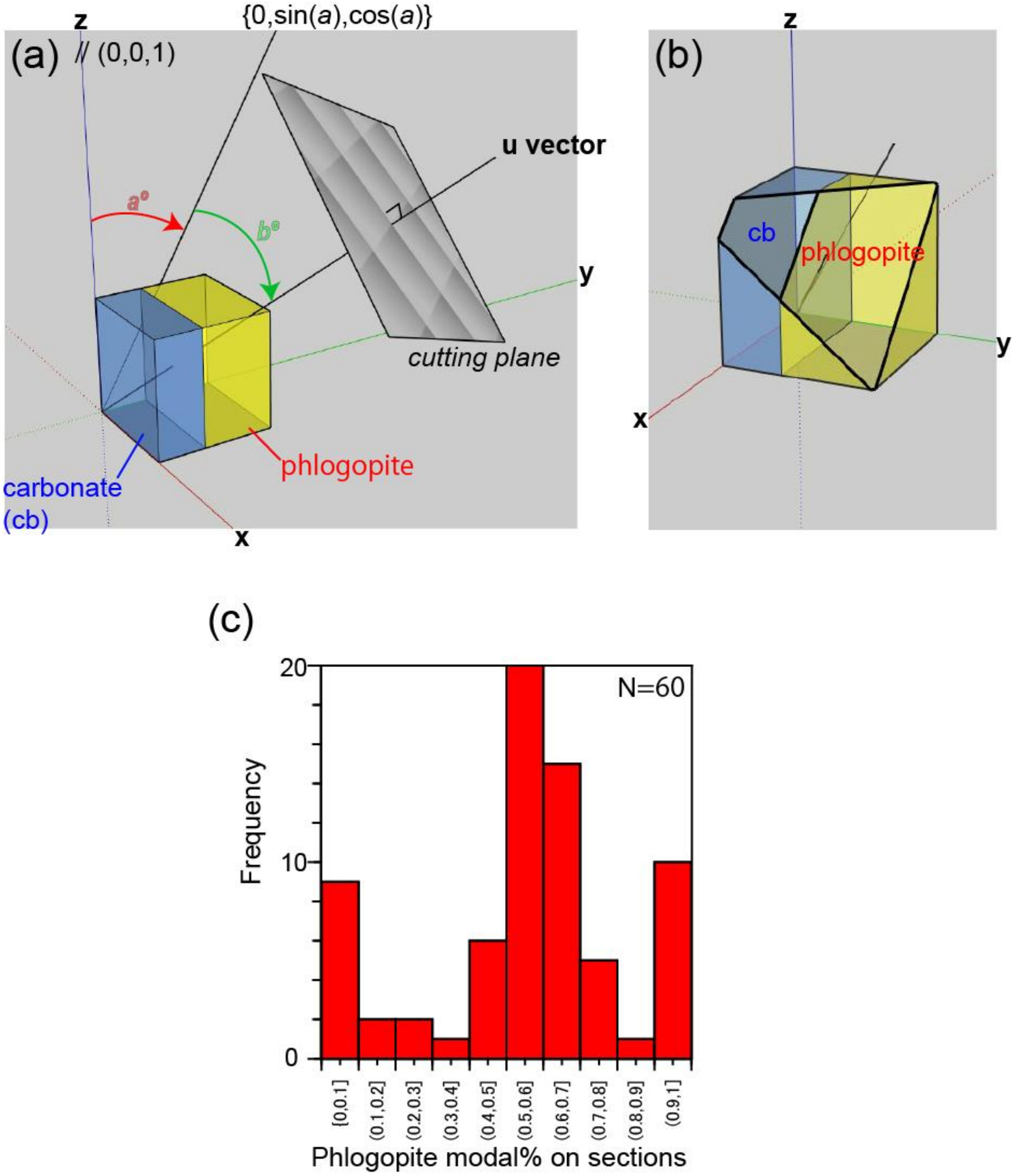

Supplementary Figure S4. Panel (a) shows a cube shaped multiphase solid inclusion (1×1×1) consisting of 60% phlogopite with a cuboid shape (1×0.6×1) and the remaining part filled by carbonate (Cb[16]). Vector **u** was produced by rotation of (0, 0, 1) vector around the x-axis by an amount (*a*°), followed by rotation around y-axis by an amount (*b*°). Cutting plane is perpendicular to the vector **u**. Panel (b) is an example slice of MSIs by a cutting plane perpendicular to the vector **u**. For each section, we measured phlogopite areal ratio. Panel (c) is a histogram of areal ratio of phlogopite on sections of a MSI for 60 times random cuts. The average of phlogopite modal% locates near 0.6. Peaks at ≅0 and 1 suggest random sections frequently intersect either through phlogopite or carbonate in high probability.

3. Supplementary application of the phase rule to multiphase solid inclusions

MSIs contain trace-element-enriched minerals. Among the 24 phases, 12 phases [various sulphides/sulphates (maucherite, orcelite, millerite, barium sulphide, pentrandite and galena as listed in Supplementary Table S3), monazite (REE), strontianite (Sr), burbankite (REE), U–Th oxide, xenotime (Y), and koragoite (W)] are rich in trace elements, and graphite formed by the reduction of carbon-dioxide. Therefore, only 11 solid phases (phlogopite, apatite, dolomite, calcite, magnesite, northethite, baryt-calcite, witherite, rutile, priderite, and spinel) and a hypothetical residual fluid are present in the 10-component system (SiO_2–TiO_2–Al_2O_3–Cr_2O_3–FeO–MgO–CaO–BaO–K_2O–CO_2–H_2O). According to the phase rule, the degree of freedom of the system (f) is calculated as: $f = 10 + 2 – 12 = 0$. Therefore, 11 solid phases can coexist with a fluid at an invariant point.

4. **Supplementary Figure S5 — comparison of pressure temperature paths of mantle wedge peridotites from high-pressure metamorphic terranes.**

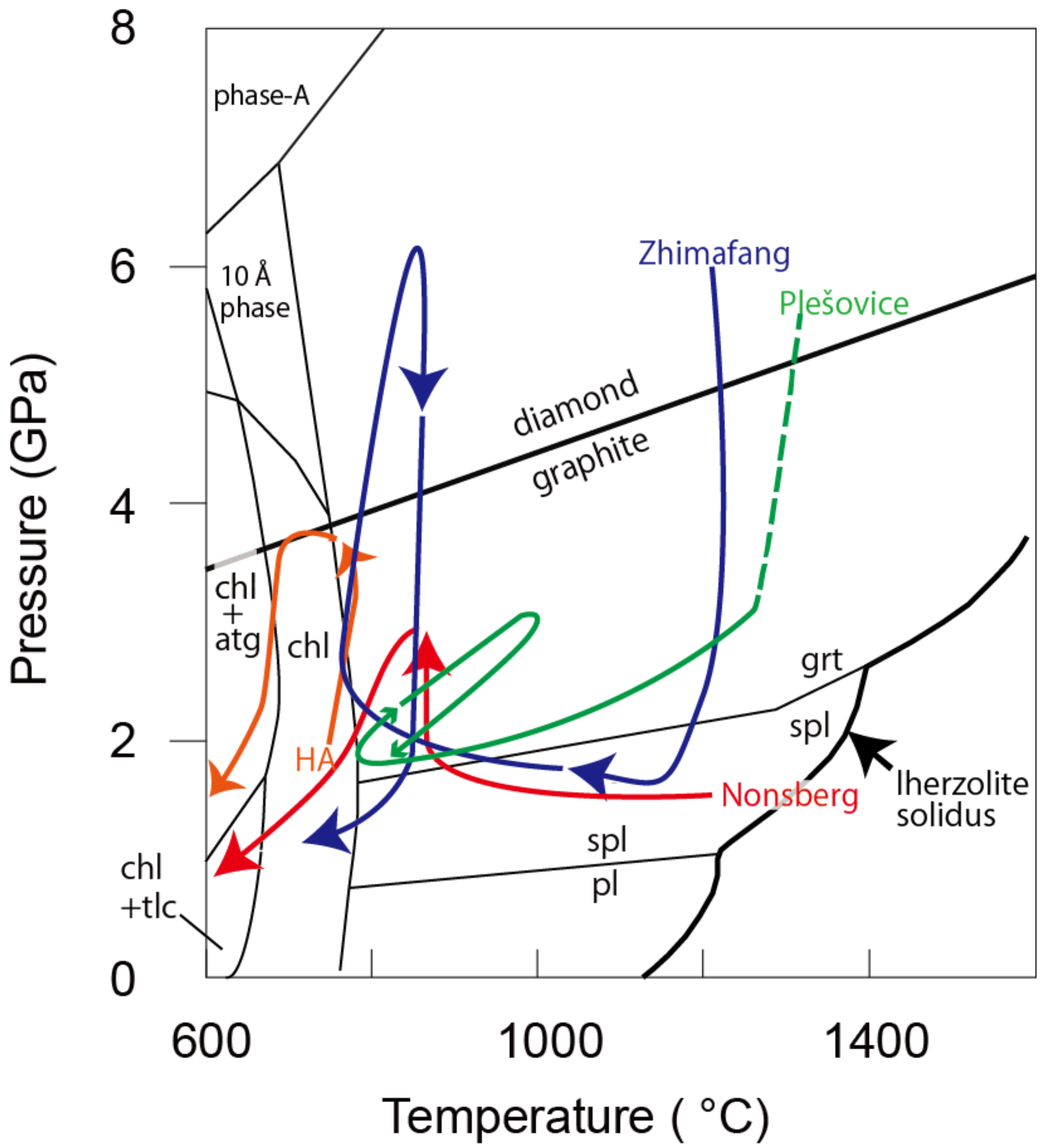

Supplementary Figure S5. Pressure-Temperature (*P-T*) paths of Plešovice peridotite (this study), Zhimafang peridotite from the Chinese Su-Lu terrane[8], Nonsberg peridotite from the Western Alps[43], and Hirashi-Akaishi (HA) garnet peridotite massif, Sanbagawa belt[45,46]. Phase boundaries are from Fig. 1, and from [7] and references therein. The *P-T* path of the Zhimafang peridotite records a prograde chlorite-stability field as proposed by [8]. chl=chlorite, atg=antigorite, tlc=talc, pl=plagioclase, spl=spinel, grt=garnet.

5. Supplementary Tables

Supplementary Table S1 | Major element compositions (weight percent) of minerals and melts.

**Mineral:**	**orthopyroxene**			**garnet**	**garnet**	**chromite**	**chromite**	**clinopyroxene**		**CPS melt**	**carbonatite**	**phlogopite**	**phlogopite**	**apatite**	**apatite**	**amphibole**
Classification/ Stage	Stage 1	Stage 3	Stage 4	Stage 2	Stage 3	Stage 1	Stage 3	Stage 2	Stage 3	MSI	calculated	MSI	Stage 3	MSI	Stage 3	Stage 3
	n=1	n=1	n=1	n=5	n=5	n=7	n=7	n=3	n=3	n=243		n=50	n=23	n=18	n=31	n=1
weight %																
SiO_2	55.4 (2)	57.2 (2)	56.9 (2)	42.13 (20)	42.01 (36)	0.04 (4)	0.02 (4)	54.4 (1)	54.7 (1)	19.57 (80)	0.1 (17)	37.11 (22)	38.67 (49)	0.09 (1)	0.19 (10)	44.4 (2)
P_2O_5	n.d.	n.d.	n.d.	n.d.	n.d.	n.d.	n.d.	n.d.	n.d.	1.63 (23)	3.4 (5)	n.d.	n.d.	40.81 (23)	40.48 (113)	n.d.
TiO_2	<0.01	0.50 (3)	0.04 (2)	0.01 (1)	0.11 (1)	0.04 (3)	0.07 (3)	<0.004	0.06 (1)	0.72 (6)	0.18 (15)	1.21 (8)	0.69 (32)	n.d.	n.d.	0.32 (2)
Al_2O_3	2.87 (4)	1.53 (3)	1.77 (3)	20.30 (10)	20.94 (13)	21. 3 (16)	22.7 (9)	1.15 (0)	2.14 (3)	9.44 (39)	<0.8	18.01 (17)	16.63 (39)	n.d.	n.d.	13.3 (2)
Cr_2O_3	0.69 (3)	0.41 (2)	0.04 (2)	4.24 (14)	3.78 (17)	49.2 (17)	47.1 (7)	0.59 (3)	1.17 (4)	1.12 (5)	<0.1	2.14 (9)	1.03 (10)	n.d.	n.d.	1.9 (3)
FeO*	5.91 (7)	5.71 (7)	7.72 (8)	7.48 (18)	6.72 (6)	19.5 (7)	19.0 (6)	1.89 (3)	2.32 (15)	1.32 (3)	0.7 (1)	1.86 (4)	2.35 (11)	0.20 (2)	0.18 (7)	2.6 (1)
MnO	0.14 (2)	0.12 (1)	0.31 (2)	0.41 (4)	0.31 (1)	0.46 (14)	0.32 (7)	0.07 (1)	0.07 (1)	0.05 (1)	0.07 (1)	0.02 (1)	0.02 (2)	0.01 (1)	0.04 (2)	0.08 (5)
MgO	33.2 (1)	34.1 (1)	32.8 (1)	18.80 (14)	19.67 (10)	10.6 (5)	11.5 (3)	17.4 (1)	17.2 (4)	18.13 (39)	12.22 (82)	23.46 (11)	24.39 (22)	0.08 (2)	0.13 (23)	18.6 (2)
CaO	0.31 (2)	0.41 (1)	0.21 (1)	6.59 (12)	6.20 (7)	n.d.	n.d.	23.6 (1)	21.4 (3)	16.63 (85)	35.04 (179)	0.05 (1)	0.02 (2)	53.1 (4)	53.14 (180)	11.9 (1)
Na_2O	0.02 (1)	0.013 (1)	<0.01	0.01 (1)	0.02 (1)	n.d.	n.d.	0.35 (2)	0.94 (2)	0.04 (1)	0.05 (1)	0.04 (1)	0.57 (14)	0.27 (6)	0.09 (8)	2.22 (6)
K_2O	0.01 (1)	0.020 (2)	0.012 (7)	n.d.	n.d.	n.d.	n.d.	n.d.	n.d.	5.18 (21)	<0.5	9.81 (6)	8.29 (47)	n.d.	n.d.	1.2 (1)
BaO	n.d.	n.d.	n.d.	n.d.	n.d.	n.d.	n.d.	n.d.	n.d.	1.46 (12)	1.2 (3)	1.7 (1)	2.68 (69)	n.d.	n.d.	0.13 (9)
SrO	n.d.	n.d.	n.d.	n.d.	n.d.	n.d.	n.d.	n.d.	n.d.	0.43 (4)	0.9 (1)	n.d.	n.d.	0.84 (14)	1.70 (103)	n.d.
F	n.d.	n.d.	n.d.	n.d.	n.d.	n.d.	n.d.	n.d.	n.d.	0.09 (1)	0.17 (3)	0.02 (1)	0.17 (21)	2.30 (15)	1.14 (73)	0.1 (1)
Cl	n.d.	n.d.	n.d.	n.d.	n.d.	n.d.	n.d.	n.d.	n.d.	0.03 (1)	0.02 (1)	0.04 (1)	0.05 (2)	0.32 (18)	1.49 (137)	0.05 (1)
H_2O	n.d.	n.d.	n.d.	n.d.	n.d.	n.d.	n.d.	n.d.	n.d.	2.21 (9)	0.02 (20)	4.19 (1)	4.13 (15)	0.57 (5)	0.83 (28)	
CO_2	n.d.	n.d.	n.d.	n.d.	n.d.	n.d.	n.d.	n.d.	n.d.	22.09 (114)	46.6 (24)	n.d.	n.d.	n.d.	n.d.	n.d.
SO_3	n.d.	n.d.	n.d.	n.d.	n.d.	n.d.	n.d.	n.d.	n.d.	0.12 (5)	0.25 (10)	n.d.	n.d.	n.d.	n.d.	n.d.
O=F, Cl	n.d.	n.d.	n.d.	n.d.	n.d.	n.d.	n.d.	n.d.	n.d.	-0.04	-0.08	-0.02	-0.07	-1.04	-0.82	
Total	100.00	99.76	99.76	100.00	99.76	101.10	100.80	99.44	99.44	100.22	98.31	100.50	99.80	97.52	99.64	99.64

Errors, which depict the variability in the different grains and multiphase solid inclusions (MSIs), are represented as the standard error (S.E.) in decimal representation, FeO* assumes a ferrous state of iron, n.d. = not determined. CPS melt=carbonated potassic silicate melt.

Supplementary Table S2 | Trace element compositions (ppm) of minerals and melts.

Mineral:	orthopyroxene			garnet	garnet	chromite	chromite	clinopyroxene		CPS melt	carbonatite	phlogopite	phlogopite	apatite	apatite	amphibole
Classification	Stage 1	Stage 3	Stage 4	Type A	Type B	Stage 1	Stage 3	Stage 2	Stage 3	MSI	calculated	MSI	Stage 3	MSI	Stage 3	Stage 3
ppm	*n=1*	*n=1*	*n=1*	*n=5*	*n=5*	*n=7*	*n=7*	*n=1*	*n=1*	*calculated*	*calculated*	*n=3*	*n=5*	*n=3*	*n=7*	*n=1*
Na	310 (33)	270 (49)	*13 (8)*	3.6 (4)	65 (12)	n.d.	n.d.	2550 (120)	6680 (270)	160 (90)	280 (190)	48 (21)	*4600 (1250)*	*1990 (430)*	*668 (593)*	*16469*
Cs	n.d.	n.d.	n.d.	n.d.	n.d.	n.d.	n.d.	n.d.	n.d.	79 (13)	98 (32)	62 (14)	50 (6)	n.d.	n.d.	<0.02
Rb	0.98 (7)	0.02 (1)	0.06 (1)	<0.1	<0.1	n.d.	n.d.	<0.1	<0.1	280 (80)	110 (230)	440 (140)	410 (79)	n.d.	n.d.	7.5 (14)
Ba	15.7 (15)	0.03 (1)	0.04 (3)	<0.1	<0.1	n.d.	n.d.	1.4 (2)	0.5 (1)	9900 (2400)	9700 (5200)	10100 (1000)	22000 (5100)	530 (51)	110 (73)	1040 (34)
Pb	0.10 (2)	0.08 (7)	0.06 (5)	<0.05	<0.05	n.d.	n.d.	50 (2)	29 (1)	32 (13)	67 (27)	0.35 (23)	*55 (14)*	21 (6)	75 (21)	18 (1)
Th	0.08 (1)	0.08 (4)	0.017 (4)	0.23 (2)	0.09 (2)	n.d.	n.d.	0.97 (50)	1.3 (4)	31 (17)	66 (36)	n.d.	n.d.	60 (34)	190 (53)	2.9 (3)
U	0.014 (2)	0.025 (4)	0.024 (2)	1.1 (1)	0.20 (6)	n.d.	n.d.	0.50 (3)	0.49 (3)	9 (5)	20 (10)	n.d.	n.d.	15 (8)	85 (24)	1.0 (1)
K	n.d.	n.d.	n.d.	n.d.	n.d.	n.d.	n.d.	n.d.	n.d.	*43000 (2000)*	*<4000*	*81400 (500)*	*69000 (3902)*	n.d.	n.d.	12000 (674)
Ta	0.003 (2)	0.024 (6)	0.014 (4)	0.015 (3)	0.018 (2)	n.d.	n.d.	0.03 (1)	0.09 (1)	29 (21)	56 (45)	5.9 (47)	0.9 (2)	n.d.	n.d.	0.39 (7)
Nb	0.04 (1)	0.076 (9)	0.14 (2)	0.27 (2)	0.24 (3)	n.d.	n.d.	0.23 (3)	0.36 (4)	830 (690)	1580 (1460)	140 (110)	7 (2)	n.d.	n.d.	4.1 (5)
La	0.14 (3)	0.07 (2)	0.035 (6)	0.28 (3)	0.06 (1)	n.d.	n.d.	33.0 (0.5)	16.0 (4)	680 (280)	1430 (580)	n.d.	n.d.	1910 (830)	2250 (497)	32 (2)
Ce	0.53 (12)	0.28 (6)	0.043 (7)	3.3 (3)	0.51 (12)	n.d.	n.d.	97 (2)	54 (1)	1870 (740)	3960 (1560)	n.d.	n.d.	6450 (2770)	4300 (1572)	108 (9)
Pr	0.10 (2)	0.050 (8)	0.003 (1)	1.0 (1)	0.13 (3)	n.d.	n.d.	12.7 (3)	8.5 (2)	200 (70)	420 (150)	n.d.	n.d.	770 (290)	440 (190)	18.4 (15)
Sr	4.7 (9)	2.6 (7)	0.043 (7)	0.54 (6)	0.22 (6)	n.d.	n.d.	610 (10)	590 (9)	*3630 (310)*	*7650 (640)*	1.9 (9)	430 (120)	12700 (5300)	16600 (1983)	1090 (40)
Nd	0.54 (10)	0.29 (6)	0.009 (6)	7.7 (10)	0.55 (12)	n.d.	n.d.	43 (1)	35 (1)	680 (230)	1440 (490)	n.d.	n.d.	2700 (1000)	1440 (690)	88 (7)
Sm	0.23 (4)	0.06 (2)	<0.004	1.7 (4)	0.25 (4)	n.d.	n.d.	3.1 (2)	5.3 (3)	130 (30)	270 (70)	n.d.	n.d.	520 (160)	210 (117)	25 (3)
Hf	0.16 (2)	0.11 (3)	0.013 (13)	0.31 (19)	0.15 (7)	n.d.	n.d.	0.27 (4)	1.7 (3)	0.9 (7)	1.6 (15)	0.3 (1)	0.19 (4)	n.d.	n.d.	4.1 (2)
Zr	6.3 (2)	4 (2)	1.1 (8)	18 (9)	4.7 (25)	n.d.	n.d.	6.8 (3)	38 (1)	60 (40)	120 (80)	7 (3)	8 (1)	2.2 (13)	1.6 (4)	138 (11)
Eu	0.058 (9)	0.011 (3)	<0.002	0.23 (6)	0.12 (2)	n.d.	n.d.	0.27 (3)	0.77 (6)	23 (5)	50 (11)	n.d.	n.d.	92 (24)	30 (16)	6.2 (6)
Ti	120 (5)	440 (14)	420 (16)	90 (6)	704 (49)	390 (70)	880 (170)	60 (7)	460 (20)	*4320 (360)*	*1080 (910)*	*7240 (450)*	*4140 (1920)*	n.d.	n.d.	1260 (91)
Gd	0.37 (6)	0.037 (12)	0.023 (10)	0.52 (13)	0.72 (12)	n.d.	n.d.	0.9 (1)	2.2 (2)	70 (20)	150 (40)	n.d.	n.d.	300 (80)	110 (50)	20 (2)
Tb	0.040 (7)	0.003 (2)	0.014 (3)	0.04 (1)	0.22 (3)	n.d.	n.d.	0.04 (1)	0.15 (2)	7 (2)	14 (4)	n.d.	n.d.	28 (8)	9 (4)	2.6 (2)
Dy	0.18 (3)	0.011 (5)	0.22 (1)	0.34 (4)	2.12 (21)	n.d.	n.d.	0.05 (1)	0.37 (7)	23 (7)	50 (14)	n.d.	n.d.	90 (27)	32 (15)	11 (1)
Ho	0.020 (5)	0.003 (1)	0.098 (8)	0.10 (1)	0.63 (4)	n.d.	n.d.	0.009 (4)	0.05 (1)	3 (1)	7 (2)	n.d.	n.d.	13 (4)	5.0 (26)	1.5 (1)
Y	0.60 (7)	0.07 (2)	2.9 (2)	2.7 (1)	16 (1)	n.d.	n.d.	0.07 (3)	0.92 (12)	80 (20)	170 (50)	0.2 (4)	*0.3 (1)*	300 (95)	130 (59)	41 (3)
Er	0.030 (7)	0.007 (4)	0.56 (4)	0.44 (2)	2.3 (2)	n.d.	n.d.	0.04 (2)	0.10 (3)	9 (3)	19 (7)	n.d.	n.d.	31 (12)	13 (8)	2.5 (3)
Tm	0.004 (1)	0.0013 (7)	0.14 (1)	0.08 (1)	0.39 (3)	n.d.	n.d.	0.002 (2)	0.006 (4)	1.4 (5)	3 (1)	n.d.	n.d.	4.2 (17)	1.5 (11)	0.22 (3)
Yb	0.017 (6)	0.010 (7)	1.50 (5)	0.80 (6)	3.4 (3)	n.d.	n.d.	0.009 (8)	0.03 (2)	6.3 (35)	13 (7)	n.d.	n.d.	18 (11)	9 (7)	1.4 (1)
Lu	0.003 (1)	0.0018 (8)	0.33 (2)	0.16 (1)	0.56 (6)	n.d.	n.d.	<0.006	0.002 (2)	0.9 (6)	2.0 (1.2)	n.d.	n.d.	2.3 (15)	1.2 (9)	0.14 (2)
Ni	1300 (33)	1090 (29)	980 (22)	25 (2)	46 (1)	180 (30)	400 (50)	360 (16)	338 (18)	2160 (510)	1430 (1220)	2820 (510)	1450 (140)	n.d.	n.d.	750 (46)
Zn	n.d.	n.d.	n.d.	n.d.	n.d.	360 (10)	2520 (610)	n.d.	n.d.	n.d.	n.d.	n.d.	n.d.	n.d.	n.d.	n.d.
Mn	n.d.	n.d.	n.d.	n.d.	n.d.	3400 (300)	2485 (190)	n.d.	n.d.	n.d.	n.d.	100 (20)	78 (8)	n.d.	n.d.	320 (14)
V	n.d.	n.d.	n.d.	285 (9)	219 (6)	1945 (130)	2220 (230)	138 (5)	221 (7)	n.d.	n.d.	510 (80)	260 (31)	n.d.	n.d.	425 (36)

Errors, which depict the variability in the different grains and multiphase solid inclusions (MSIs), are represented as the standard error (S.E.) in decimal representation. Italic values with underlines are EPMA analyses, n.d. = not determined.

Supplementary Table S3 | Volume percent of minerals in multiphase solid inclusions of Plešovice peridotite.

		Type A peridotite		Type B peridotite		All samples	
		MSI	S.E.	MSI	S.E.	MSI	S.E.
Daughter minerals in Multiphase solid inclusions	volume %	n=186		n=57		n=243	
	phlogopite	54.36	2.37	48.59	4.93	51.11	2.13
	apatite	2.14	0.39	5.47	1.74	2.72	0.48
	graphite	1.33	0.32	0.71	0.31	1.14	0.25
	dolomite	27.11	2.17	24.95	4.06	25.75	1.87
	calcite	12.34	1.62	19.33	3.66	13.12	1.42
	magnesite	0.62	0.54	–	–	0.47	0.41
	norsethite	0.61	0.23	–	–	0.45	0.17
	baryt-calcite	0.24	0.09	0.07	0.07	0.20	0.07
	witherite	0.01	0.01	0.03	0.03	0.01	0.01
	strontianite	0.07	0.04	0.06	0.06	0.06	0.03
	burbankite	0.02	0.02	–	–	0.02	0.01
	monazite	0.73	0.18	0.23	0.15	0.58	0.14
	U-Th oxide	0.02	0.01	0.05	0.02	0.03	0.01
	xenotime	0.00	0.00	–	–	0.00	0.00
	rutile	0.05	0.05	0.04	0.03	0.04	0.03
	koragoite	–	–	0.04	0.04	0.01	0.01
	maucherite	0.02	0.01	0.02	0.01	0.02	0.01
	orcelite	–	–	–	–		
	millerite	0.06	0.04	0.10	0.08	0.07	0.03
	barium sulfide	0.011	0.008	0.10	0.10	0.03	0.02
	pentrandite	0.02	0.01	0.18	0.15	0.05	0.03
	galena	0.22	0.09	0.04	0.04	0.17	0.06
SDM	spinel	0.58	0.25	0.58	0.28	0.49	0.16
	Cr priderite	0.01	0.01	0.03	0.02	0.01	0.01
secondary minerals	chlorite	1.79	0.86	3.46	1.18	1.54	0.36
	talc	0.34	0.20	0.14	0.14	0.24	0.13
	serpentine	1.72	0.50	1.19	0.71	1.25	0.31
	diaspore	0.32	0.23	–	–	0.19	0.13
	brucite	0.15	0.09	–	–	0.10	0.06

MSIs = multiphase solid inclusions; S.E. = standard error of volume %; "-" represents the null volume of a mineral in MSIs; SDM=Step daughter mineral[24] that crystallizes as a reaction phase between melt and host chromite; Type A peridotite samples used are PQ 101 (a, b, l, q), and Type B peridotite samples used are PQ 201, 207, 210, 211, 212.
